# TMEM70 and TMEM242 help to assemble the rotor ring of human ATP synthase and interact with assembly factors for complex I

**DOI:** 10.1073/pnas.2100558118

**Published:** 2021-03-22

**Authors:** Joe Carroll, Jiuya He, Shujing Ding, Ian M. Fearnley, John E. Walker

**Affiliations:** ^a^Medical Research Council Mitochondrial Biology Unit, University of Cambridge, Cambridge CB2 0XY, United Kingdom

**Keywords:** human mitochondria, ATP synthase, assembly, TMEM70, TMEM242

## Abstract

Human mitochondrial ATP synthase is a molecular machine with a rotary action bound in the inner organellar membranes. Turning of the rotor, driven by a proton motive force, provides energy to make ATP from ADP and phosphate. Among the 29 component proteins of 18 kinds, ATP6 and ATP8 are mitochondrial gene products, and the rest are nuclear gene products that are imported into the organelle. The ATP synthase is assembled from them via intermediate modules representing the main structural elements of the enzyme. One such module is the c_8_-ring, which provides the membrane sector of the enzyme’s rotor, and its assembly is influenced by another transmembrane (TMEM) protein, TMEM70. We have shown that subunit c interacts with TMEM70 and another hitherto unidentified mitochondrial transmembrane protein, TMEM242. Deletion of TMEM242, similar to deletion of TMEM70, affects but does not completely eliminate the assembly of ATP synthase, and to a lesser degree the assembly of respiratory enzyme complexes I, III, and IV. Deletion of TMEM70 and TMEM242 together prevents assembly of ATP synthase and the impact on complex I is enhanced. Removal of TMEM242, but not of TMEM70, also affects the introduction of subunits ATP6, ATP8, j, and k into the enzyme. TMEM70 and TMEM242 interact with the mitochondrial complex I assembly (the MCIA) complex that supports assembly of the membrane arm of complex I. The interactions of TMEM70 and TMEM242 with MCIA could be part of either the assembly of ATP synthase and complex I or the regulation of their levels.

## Significance

The oxidation of energy rich compounds generates a proton motive force, a chemical potential difference for protons, across the inner membranes of the mitochondria. The proton motive force drives the turning of the rotor in the membrane domain of the ATP synthase. This rotation provides the energy to synthesize the ATP required to sustain life. The assembly in the inner organellar membrane of human ATP synthase from 27 nuclear encoded proteins and 2 mitochondrially encoded subunits involves the formation of intermediate modules representing the F_1_-catalytic domain, the peripheral stalk, and the c_8_-ring in the membrane part of the rotor. The assembly of the c_8_-ring requires the participation of two membrane-associated proteins, TMEM70 and, as we demonstrate, TMEM242.

**Fig. 1.**
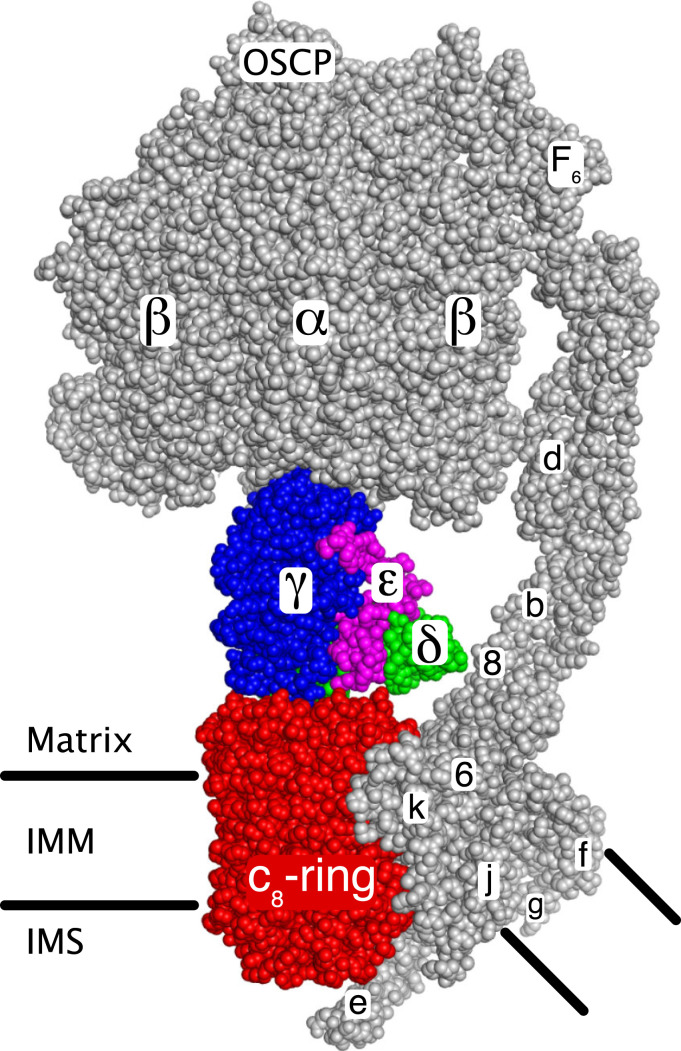
The rotor and stator components of monomeric bovine ATP synthase. The two main functional components of the enzyme, the rotor (colored) and the stator (gray), are shown. The rotor consists of the c_8_-ring (red) associated with the IMM, and the central stalk consisting of subunits γ (blue), δ (green), and ε (magenta). The upper region of the γ-subunit (view obscured) is asymmetrical and penetrates into the upper spherical part of the stator, consisting of three α-subunits and three β-subunits where the three catalytic sites of the enzyme are found. The spherical region and the central stalk together are known as the F_1_-domain. The F_1_-domain and the upper part of the associated PS on the right, extend into the mitochondrial matrix. The PS, made of the oligomycin sensitivity conferral protein (OSCP) and subunits b, d, and F_6_, is attached via the OSCP to the upper surface of the spherical region and extends into the IMM. The membrane domain of the PS is associated with subunits e, f, g, j (or 6.8-kDa proteolipid), ATP6 (or 6), and ATP8 (or 8). The membrane region consisting of subunits e, f, g and the transmembrane α-helices of subunits b and associated lipids is referred to as “the wedge” (23). Subunit k (or diabetes-associated protein in insulin sensitive tissue, DAPIT) is bound to ATP6, and subunit e extends into the intermembrane space (IMS). The turning of the rotor, driven by energy from the transmembrane proton motive force, brings about a series of structural changes in each of the three catalytic sites leading to the binding of substrates and the formation and release of three ATP molecules for each 360° rotary cycle. Image credit: M. G. Montgomery (University of Cambridge, United Kingdom).

The ATP synthase in human mitochondria provides most cellular ATP under aerobic conditions. Energy derived from oxidative metabolism generates a proton-motive force across the inner mitochondrial membrane (IMM), and the ATP synthase harnesses the proton-motive force to make ATP from ADP and phosphate by a rotary mechanism (1–3). Human ATP synthase is an assembly of 29 subunits of 18 types (including the regulatory protein, IF_1_) with a combined molecular mass of 591 kDa (3, 4). In the membrane-bound monomeric complex, the subunits are organized into the rotor and stator components of this molecular machine (Fig. 1), and the monomers form dimers arranged in long rows along the tips of the cristae in the IMM (5–8). In each monomeric complex, two membrane subunits, ATP6 and ATP8, are encoded in mitochondrial DNA (9), whereas the other subunits are nuclear encoded proteins, synthesized in the cytosol and imported into the IMM and the matrix of the organelle, where the enzyme complex is put together. Previously, we have described the later stages of the process of assembly of the human ATP synthase, including the insertion of the mitochondrially encoded subunits ATP6 and ATP8 in an unknown order into a key intermediate complex (10, 11). This intermediate consists of the F_1_-catalytic domain attached to the membrane associated c_8_-ring component of the enzyme’s rotor plus an elongated peripheral stalk (PS) complex bound to the F_1_-domain and extending into the membrane domain of the enzyme. We have shown that this intermediate can be arrived at by three independent routes, where the PS—including associated membrane subunits e, f, and g—is built by two alternative paths onto a preassembled F_1_-c_8_ ring intermediate, and the third where the c_8_-ring is added to a preassembled F_1_-PS complex.

These findings suggest that the c_8_-ring is built as a separate module before being introduced intact into these two intermediate complexes. In *Saccharomyces cerevisiae*, the F_1_-catalytic domain is assembled as another separate module, requiring the involvement of three assembly factors, ATP11, ATP12, and FMC1 (12, 13). Bovine ATPAF1 and ATPAF2, the respective orthologs of ATP11 and ATP12, were needed for the efficient production of the bovine F_1_-domain in *Escherichia coli* (14), and the human orthologs are presumed to play similar roles (15, 16). Human FMC1 is also required for assembly of human ATP synthase, and it interacts with ATPAF2 (17). Another protein assembly factor transmembrane protein 70 (TMEM70) found in the IMM influences the assembly of the c_8_-ring (18). Mutations in TMEM70 affect the levels of both ATP synthase (19), and the mitochondrial respiratory enzyme complex NADH:ubiquinone oxidoreductase, or complex I (20, 21), but even in the absence of TMEM70 both complexes are still assembled, albeit at diminished levels (22). Here, we show that the assembly of the c_8_-ring requires not only TMEM70 but also a previously unidentified transmembrane protein, TMEM242. Moreover, both TMEM70 and TMEM242 interact with subunit c, and also with the mitochondrial complex I assembly complex, or MCIA complex.

## Results

### Assembly Factors Associated with Subunit c.

**Fig. 2.**
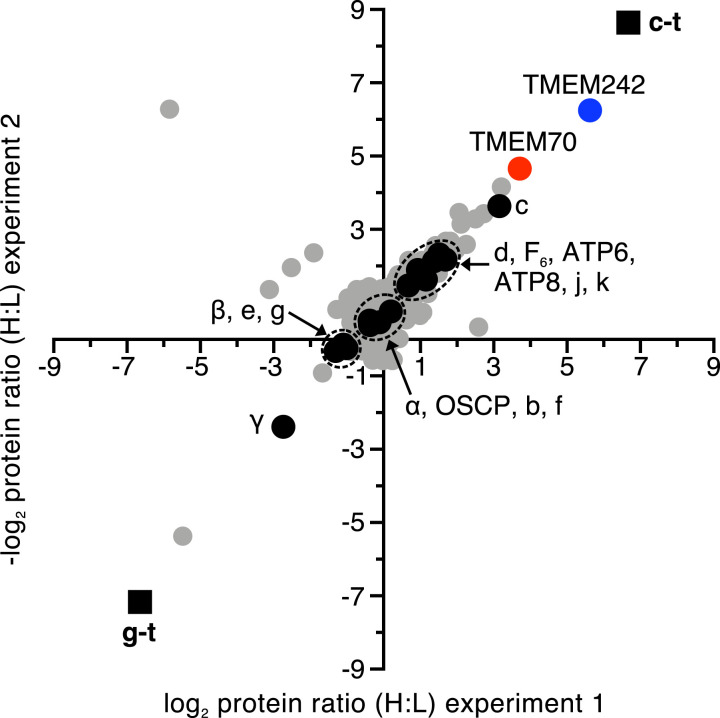
Proteins associated with ATP synthase subunits c and g in HEK293-Δδ cells. Filled black square, tagged proteins; filled black circle, ATP synthase subunits; filled red circle, TMEM70; filled blue circle, TMEM242; filled gray circle, other proteins. Subunits c-t and g-t were overexpressed separately in HEK293-Δδ Flp-In T-REx cells grown in SILAC media. Equal amounts of cells were combined, and proteins extracted from mitoplasts with digitonin (detergent:protein, 12:1, g:g). Tagged subunits and associated proteins were purified by affinity chromatography. The experiment was performed twice with reciprocal SILAC labeling: experiment 1, c-t heavy (H) label plus g-t light (L) label, and experiment 2, vice versa. Each data point corresponds to the relative abundance ratio of an identified protein from the two complementary MS analyses (Datasets S1–S3).

Subunits c and g, each with tandem C-terminal Strep II and FLAG tags, and known as c-t and g-t, respectively, were incorporated into ATP synthase in human embryonic kidney (HEK) 293 cells (*SI Appendix*, Fig. S1*A*). Subunit g is a component of the protein “wedge” in the membrane domain of the ATP synthase, but it does not interact directly with the c_8_-ring (Fig. 1) (23). Therefore, in order to investigate which protein factors might be associated with the c-subunit during its assembly into c_8_-rings, subunit c-t was introduced into HEK293-Δδ cells, where the gene for the δ-subunit of the ATP synthase had been disrupted (11). These cells are incapable of assembling the F_1_-domain of ATP synthase, but retain the ability to assemble c_8_-rings and accumulate oligomeric forms of the c-subunit (*SI Appendix*, Fig. S1*B*). In a control experiment, the g-t subunit was introduced separately into the same cells. Measurement of the relative levels of proteins associated with the tagged subunits in mitoplasts confirmed that, as expected, the c-subunit was associated with TMEM70, and also revealed the association of the c-subunit with TMEM242 (Fig. 2 and Datasets S1–S3), a membrane protein of hitherto unknown function and subcellular location. No mitochondrial proteins were found in association with the g-t subunit.

### Location and Topography of TMEM242.

**Fig. 3.**
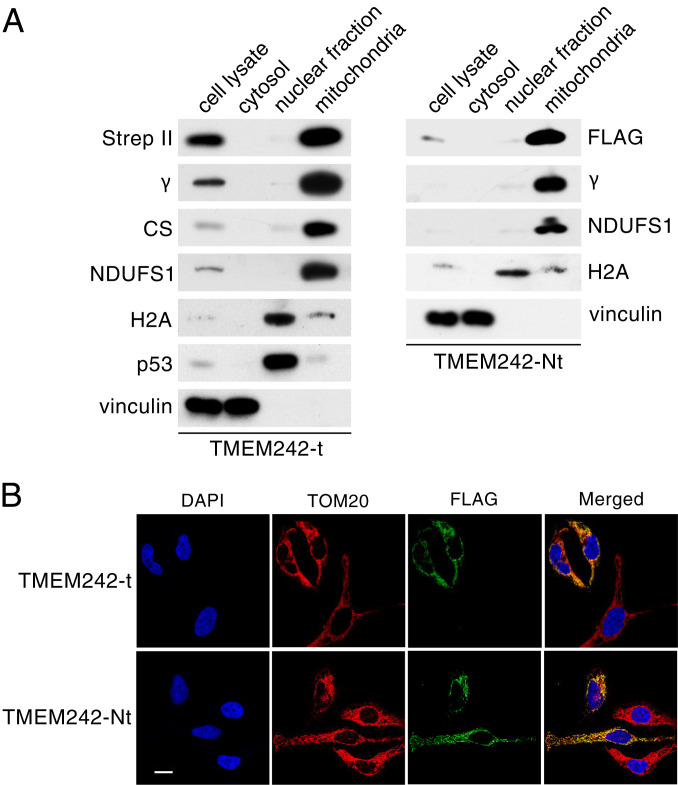
Subcellular location of TMEM242. (*A*) Analysis by SDS-PAGE of subcellular fractions (*Left*) from HEK293 cells expressing TMEM242 with tandem C-terminal Strep II and FLAG tags (TMEM242-t), and HeLa cells expressing TMEM242 with an N-terminal FLAG tag (TMEM242-Nt) (*Right*). Subcellular fractions are indicated above the panels. Proteins were detected with antibodies indicated on the left and right against FLAG and Strep II tags, the γ-subunit of ATP synthase, citrate synthase (CS), subunit NDUFS1 of complex I, histone H2A, p53 and vinculin; (*B*) immunofluorescence microscope images of HeLa cells expressing transiently (*Upper*) TMEM242-t and (*Lower*) TMEM242-Nt. (*Upper*) Two cells expressing TMEM242-t and an untransformed cell; (*Lower*) two cells expressing TMEM242-Nt and two untransformed cells; blue, nuclei; red, TOM20; green, TMEM242. All images were taken with the same magnification (scale bar, 20 μm).

In order to study the subcellular location of TMEM242, the protein with tandem C-terminal Strep II and FLAG tags (TMEM242-t) was expressed in HEK293 cells, and the protein with an N-terminal FLAG tag (TMEM242-Nt) was expressed in HeLa cells. Examination of the subcellular fractions demonstrated that TMEM242 localizes uniquely to the mitochondria (Fig. 3*A*), and the localization of TMEM242 to mitochondria in HeLa cells was confirmed by immunofluorescence microscopy (Fig. 3*B*). TMEM242 was not removed from HEK293 mitochondria by washing them at alkaline pH, but it was extracted with buffer containing deoxycholate concentrations of 0.5% or greater, as were the known IMM components subunit c and ATAD3 (ATPases associated with diverse cellular activities domain-containing protein 3) (*SI Appendix*, Fig. S2 *A* and *B*). Therefore, TMEM242 is an integral component of the human IMM. By trypsinolysis of intact and lysed mitochondria from HEK293 cells, it was demonstrated that the N- and C-terminal regions of TMEM242 are in the matrix (*SI Appendix*, Fig. S2 *C*–*E*).

### Removal of TMEM70 and TMEM242 from Human Cells.

**Fig. 4.**
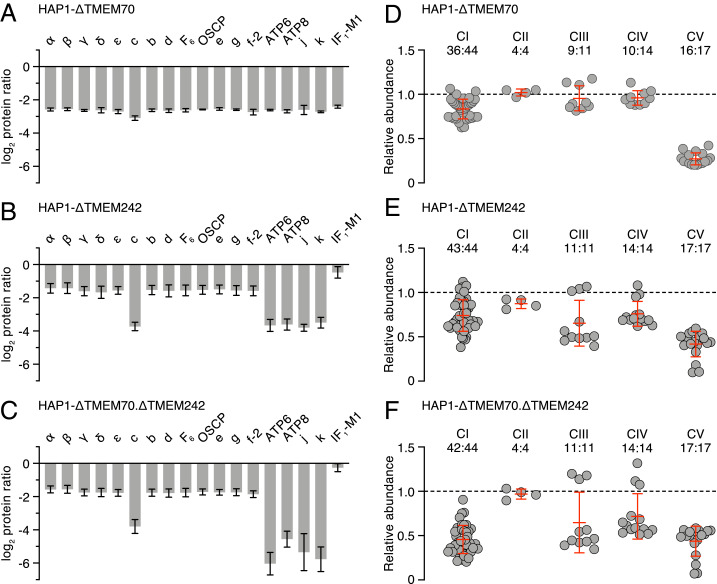
Impact of deletion of TMEM70, TMEM242, or TMEM70 and TMEM242 together, on the composition of ATP synthase and respiratory chain complexes. (*A*–*C*) Relative abundances of subunits and the inhibitor protein IF_1_ in ATP synthase immunopurified from mitoplasts of HAP1-ΔTMEM70, -ΔTMEM242, and -ΔTMEM70.ΔTMEM242 cells, respectively. IF_1_-M1 is a specific mature form of IF_1_ (10); f-2, is an isoform of subunit f (UniProt P56134). The median values of both relative abundance ratios for proteins derived from MS analysis in two complementary SILAC-labeling experiments are given; error bars show the range of the values. (*D*–*F*) Relative abundances of subunits of complexes I to IV (CI–CIV) and ATP synthase (CV), excluding IF_1_, in extracts of mitoplasts from HAP1-ΔTMEM70, -ΔTMEM242, and -ΔTMEM70.ΔTMEM242 cells, respectively; the points represent the median of both relative abundance values of subunits in complementary experiments normalized to citrate synthase. Error bars are the mean of subunit abundances ±SD. The ratio above each complex is the number of subunits identified from CI to CV to their total subunits. Protein ratios were derived from a minimum of two peptide ratios, except for subunit c in both experiments in *A*, and ATP6 experiment 1 in *B*, where the values are derived from a single peptide ratio. In *D*, no ratio was obtained for subunit c. See Datasets S4–S20.

The genes *TMEM70* and *TMEM242* were disrupted in HAP1 cells, both singly and together, producing HAP1-ΔTMEM70, -ΔTMEM242, and -ΔTMEM70.ΔTMEM242 cells, and preventing expression of the targeted genes in all three cases (*SI Appendix*, Figs. S3–S5 and Tables S1 and S2). The removal of TMEM70 diminished the content of ATP synthase in mitochondria, with a similar effect on all subunits of the enzyme (Fig. 4 *A* and *D*, *SI Appendix*, Fig. S6, and Datasets S4–S9), but, as noted before (18, 22), its removal did not abolish the assembly of ATP synthase completely. The removal of TMEM242 had a more specific effect than the removal of TMEM70. Again, in both the purified enzyme and in mitoplasts, the overall content of ATP synthase was reduced, but also the levels of membrane subunits c, ATP6, ATP8, j, and k in the purified complex were diminished to a greater extent than the other subunits of the complex (Fig. 4 *B* and *E*, *SI Appendix*, Fig. S6, and Datasets S10–S15). Thus, in HAP1-∆TMEM242 cells, two different assemblages containing subunits of ATP synthase coexist, with the F_1_-PS-e-f-g subcomplex predominating in the presence of minor amounts of the intact enzyme. The F_1_-PS-e-f-g subcomplex is the vestigial complex that remains after deletion of subunit c in HAP1-∆c cells (4). The removal of TMEM70 and TMEM242 together mirrored the effect of removal of TMEM242 alone in decreasing the levels of subunits of ATP synthase, but with an even greater pronounced selective effect on diminishing subunits ATP6, ATP8, j, and k in purified vestigial ATP synthase complexes, and ATP6, ATP8, and j in mitoplasts (Fig. 4 *C* and *F*, *SI Appendix*, Fig. S6, and Datasets S16–S20). Consistent with these analyses, minor residual levels of subunits c, ATP8, and j were immuno-detected in purified ATP synthase complexes from HAP1-ΔTMEM242 cells, but subunits c, ATP8, and j were not detected in similar samples from HAP1-ΔTMEM70.ΔTMEM242 cells (*SI Appendix*, Fig. S7 *A* and *B*).

**Fig. 5.**
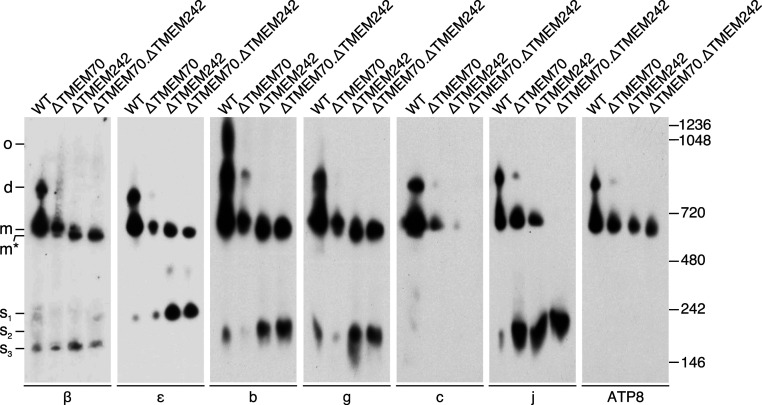
Oligomeric states of ATP synthase and vestigial complexes in HAP1 cells devoid of TMEM70 and TMEM242. CN-PAGE analysis of digitonin extracts (detergent:protein, 10:1, g:g) of mitoplasts from HAP1-WT, HAP1-∆TMEM70, HAP1-∆TMEM242, and HAP1-∆TMEM70.∆TMEM242 cells. Below each panel is shown the specificity of the antibody for an individual subunit of ATP synthase employed to detect the complexes related to ATP synthase indicated on the left; d, dimers; m, monomers; m*, the F_1_-PS-e-f-g complex; o, oligomers; s_1_, incompletely characterized subcomplex containing the ε-subunit; s_2_, subcomplex of subunits b, e, f, and g, and of a second subcomplex containing subunit j; s_3_, incompletely characterized subcomplex containing the β-subunit. The positions of molecular mass markers (kDa) are shown on the right. The total protein in the sample from HAP1-∆TMEM70 cells was twice that from HAP1-WT cells, and those from HAP1-∆TMEM242 and HAP1-∆TMEM70.∆TMEM242 cells were three times greater than HAP1-WT cells. For complementary data, see *SI Appendix*, Fig. S8.

HAP1-ΔTMEM70 cells contained both the monomeric and dimeric intact ATP synthase, albeit at diminished levels relative to HAP1-WT cells, plus lesser amounts of vestigial complexes of ATP synthase (Fig. 5 and *SI Appendix*, Fig. S8). In the HAP1-ΔTMEM242 cells a small amount of the intact monomer was present, plus significant levels of the F_1_-PS-e-f-g subcomplex (Fig. 5 and *SI Appendix*, Fig. S8), which was detected also by mass spectrometry (MS) analysis (Fig. 4*B*). No dimeric ATP synthase was detected in HAP1-ΔTMEM242 cells, and subunits c, j, and ATP8 were found only in the intact monomer (Fig. 5). In addition, there was an increased level of the assembly intermediate subcomplex, b-e-g-f (11), and two incompletely characterized subcomplexes were present; one, containing subunit j, migrated to a similar position to the b-e-g-f subcomplex, and the other, a 250-kDa subcomplex, contained the central stalk components, subunits δ and ε (Fig. 5 and *SI Appendix*, Fig. S8). The removal together of TMEM70 and TMEM242 led to the formation of mainly the F_1_-PS-e-f-g subcomplex (Fig. 5 and *SI Appendix*, Fig. S8), and a small amount of the same subcomplex plus ATP8 (Figs. 4 and 5). In addition, the smaller subcomplexes (s_1_ and s_2_ in Fig. 5), observed also in the ΔTMEM242 cells, were detected, but there was no indication of intact monomeric ATP synthase.

As reported previously (22), the removal of TMEM70 alone had a greater effect on the ATP synthase than on complex I, but the content of complex I was diminished relative to WT cells, whereas the contents of subunits of complexes II, III, and IV were not affected significantly (Fig. 4*D* and Datasets S6–S9). In contrast, in HAP1-ΔTMEM242 and HAP1-ΔTMEM70.ΔTMEM242 cells, complexes I, III, and IV were all reduced (Fig. 4 *E* and *F*, *SI Appendix*, Fig. S7*C*, and Datasets S13–S15 and S18–S20). These effects of deleting TMEM70 and TMEM242, both singly and together, on the content of ATP synthase and respiratory complexes I, III, and IV were reflected in a progressive decrease in oxygen consumption and loss of oligomycin-sensitive inhibition in HAP1-ΔTMEM70, HAP1-ΔTMEM242, and HAP1-ΔTMEM70.ΔTMEM242 cells (*SI Appendix*, Fig. S9). In comparison with HAP1-WT cells, the rates of extracellular acidification by HAP1-ΔTMEM70, HAP1-ΔTMEM242, and HAP1-ΔTMEM70.ΔTMEM242 cells were greater under uninhibited basal conditions, and cell growth was slower (*SI Appendix*, Fig. S9).

### Roles of TMEM70 and TMEM242.

**Fig. 6.**
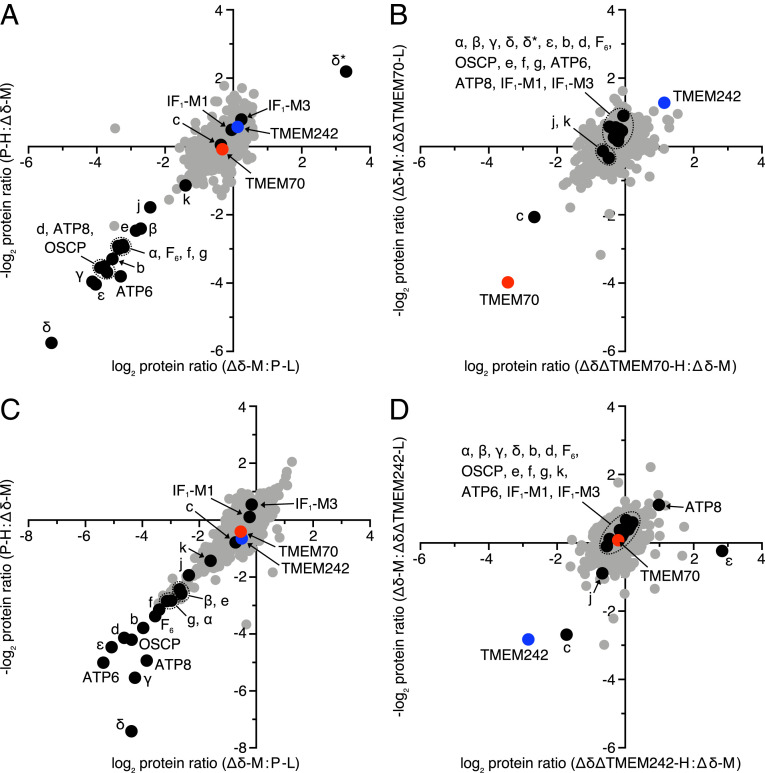
Deletion of TMEM70 and TMEM242 in cells lacking the δ-subunit of human ATP synthase. (*A* and *B*) The relative protein abundances determined by SILAC and MS analysis in mitoplasts from HEK293-Δδ cells relative to HEK293 parental (P) cells (*A*), and from HEK293-Δδ.ΔTMEM70 cells relative to HEK293-Δδ cells (*B*). (*C* and *D*) The protein abundances in mitoplasts from HEK293-Δδ cells relative to HEK293-P cells (*C*), and of HEK293-Δδ.ΔTMEM242 cells relative to HEK293-Δδ cells (*D*). Filled black circle, ATP synthase subunits; filled red circle, TMEM70; filled blue circle, TMEM242; filled gray circle, other identified proteins. Cells were subjected to three-way SILAC labeling, where they were heavy labeled (H; ^13^C_6_^15^N_4_-Arg, ^13^C_6_^15^N_2_-Lys), medium labeled (M; ^13^C_6_^14^N_4_-Arg, ^2^D_4_-Lys), and light labeled (L; ^12^C_6_^14^N_4_-Arg, ^12^C_6_^14^N_2_-Lys). Equal quantities of labeled cells were mixed in the combinations (P-L, Δδ-M, and Δδ.ΔTMEM70-H or Δδ.ΔTMEM242-H) and in a second experiment (P-H, Δδ-M, and Δδ.ΔTMEM70-L or Δδ.ΔTMEM242-L). In both experiments, the points on the scatter plot represent the log base 2 values of protein ratios. In *A* and *B*, δ* is a nonfunctional aberrant form of the δ-subunit produced in HEK293-Δδ and HEK293-Δδ.ΔTMEM70, which migrates to a different position on SDS/PAGE gels than the authentic δ-subunit (11). Except for TMEM242 in the *C* and *D* second-labeling experiment, where the values are derived from a single peptide ratio, protein ratios were derived from a minimum of two peptide ratios. See Datasets S21 and S22.

The roles of TMEM70 and TMEM242 in integrating subunit c into the IMM and in assembling c_8_-rings were investigated by studying the effects of disrupting the genes for the two assembly factors in HEK293-∆δ cells (Fig. 6, *SI Appendix*, Figs. S5 and S10, and Datasets S21 and S22). Although the HEK293-∆δ cells cannot assemble ATP synthase (11), as mentioned above, they accumulate oligomeric subunit c to a similar level to HEK293 parental cells (Fig. 6 *A* and *C*). However, relative to the HEK293-∆δ cells, the level of subunit c in both HEK293-∆δ.∆TMEM70 and HEK293-∆δ.∆TMEM242 cells had decreased significantly, whereas the levels of other subunits were unaffected, except for subunit j, which was diminished slightly in HEK293-∆δ.∆TMEM242 cells (Fig. 6 *B* and *D*). The specific decrease in the levels of subunit c in HEK293-∆δ.∆TMEM70 and HEK293-∆δ.∆TMEM242 cells was supported by immunodetection of proteins in mitoplasts (*SI Appendix*, Fig. S11). Furthermore, no subunit c was detected in mitoplasts from HEK293-∆δ.∆TMEM70.∆TMEM242 cells and the levels of two complex I subunits, NDUFS1 and NDUFB8, had decreased (*SI Appendix*, Fig. S11). As transcripts for subunit c from *ATP5MC1-3* were present in normal or higher amounts in both HEK293-∆δ.∆TMEM242 and HEK293-∆δ.∆TMEM70.∆TMEM242 cells (*SI Appendix*, Fig. S12), it is probable that the lower level of subunit c in these cells arises from the degradation of the protein. In HAP1-∆TMEM242 and HAP1-∆TMEM70.∆TMEM242 cells, the levels of transcripts were lower than in HAP1-WT cells, but were insufficient to account for the decreased levels of protein, which again probably arise from their degradation (Fig. 4 and *SI Appendix*, Figs. S7 and S12).

**Fig. 7.**
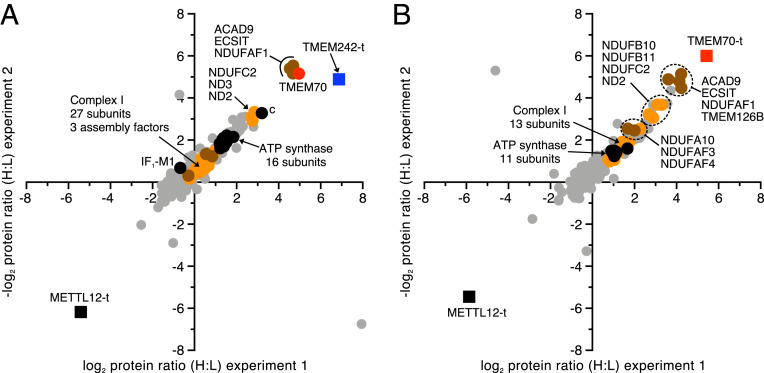
Proteins associated with TMEM242 and TMEM70. Cells labeled by SILAC were analyzed by MS. Filled blue square, TMEM242-t; filled red square, TMEM70-t; filled black square, METTL12-t; filled black circle, ATP synthase subunit; filled red circle, TMEM70; filled orange circle, complex I subunit; filled brown circle, complex I assembly factor; filled gray circle, other proteins. (*A* and *B*) TMEM242-t or TMEM70-t, respectively, were overexpressed in Flp-In T-REx HEK293 cells grown in SILAC media. Flp-In T-REx HEK293 cells expressing the mitochondrial methyltransferase METTL12 with tandem C-terminal Strep II and FLAG tags (METTL12-t) provided a negative control in both experiments. Protein ratios were derived from a minimum of two peptide ratios, except for subunit c in both experiments in *B*, where the values are derived from a single peptide ratio. All identified proteins are given in Datasets S23–S25.

When TMEM242-t and similarly tagged TMEM70 (TMEM70-t) were expressed separately in HEK293 cells, the most significantly associated proteins were ACAD9 (acyl-CoA dehydrogenase family member 9), ECSIT (evolutionarily conserved signaling intermediate in Toll pathway), and NDUFAF1 (NADH:ubiquinone oxidoreductase complex assembly factor 1), and in addition, TMEM70-t was associated with transmembrane protein 126B (TMEM126B) (Fig. 7 and Datasets S23–S25). ACAD9, ECSIT, NDUFAF1, and TMEM126B have been identified previously as components of the MCIA complex (24). TMEM242-t was also bound to TMEM70 and, to a lesser extent, to subunit c and to the NDUFC2, ND2, and ND3 membrane subunits of complex I (Fig. 7*A*). In the equivalent TMEM70-t experiment, ratios for TMEM242 and the ND3 subunit of complex I were not obtained. Subunit c was enriched slightly relative to other subunits of ATP synthase, and NDUFB10, NDUFB11, NDUFC2, and ND2, components of the membrane domain subunits of complex I, were associated significantly with TMEM70-t (Fig. 7*B* and Dataset S25).

The connection between TMEM70 and TMEM242 and components of the MCIA complex was corroborated by immunodetection of the proteins that copurified with TMEM70-t and TMEM242-t expressed individually in HEK293 cells. TMEM70-t was found in association with ACAD9, NDUFAF1, and also with an additional assembly factor for complex I, the translocase of IMM domain-containing protein 1, or TIMMDC1 (*SI Appendix*, Fig. S13*A*). In addition, subunit c was associated with TMEM70-t (*SI Appendix*, Fig. S13*A*), and TMEM242-t was bound to TMEM70, subunit c, ACAD9, and NDUFAF1, and weakly to TIMMDC1 (Fig. 7 and *SI Appendix*, Fig. S13*B*). As expected, TMEM70 was associated with subunit c-t in HEK293-∆δ cells, but ACAD9 and NDUFAF1 were not identified (Fig. 2 and *SI Appendix*, Fig. S13*C*). It was not possible to confirm by immunodetection that TMEM242 was bound to TMEM70-t, as no antibody against TMEM242 is currently available.

In native gel analyses (*SI Appendix*, Fig. S13*D*), subunit c from HEK293-∆δ cells was identified in the form of oligomers in the molecular mass range of 150 to 700 kDa. In contrast, in affinity-purified samples of TMEM70-t and TMEM242-t, subunit c was found exclusively in smaller complexes with molecular masses in the range from 60 to 150 kDa. In addition, TMEM70-t comigrated with a subunit c complex of ∼150 kDa, and TMEM242-t with other subunit c complexes of 60 to 100 kDa. TMEM70-t itself was detected in oligomers around and beyond the highest molecular mass marker of 1,236 kDa. The MCIA components NDUFAF1 and ACAD9 were present in native complexes with molecular masses ranging from 300 to 700 kDa.

## Discussion

Previously, it was known that the assembly of the c_8_-ring of human ATP synthase is influenced by TMEM70, a protein found in the IMM, but not a component of the ATP synthase itself (18, 22). Pathogenic states associated with deficiencies in ATP synthase arise most frequently from mutations in TMEM70 (19, 25, 26), consistent with the protein playing an important part in the assembly of the complex. Here, it is shown that the assembly of the c_8_-ring requires not only TMEM70 but also a second IMM protein, TMEM242. TMEM70 is conserved throughout eukarya (25), and TMEM242 is found in metazoans (*SI Appendix*, Figs. S14 and S15), and possibly slime molds, some fungi, and elsewhere. Here, we have shown that TMEM242 functions directly in the assembly of ATP synthase and that both TMEM242 and TMEM70 interact specifically with subunit c. Although there is no significant sequence relationship between TMEM70 and TMEM242, both proteins are predicted to have two transmembrane α-helices (*SI Appendix*, Fig. S16) and are oriented in a similar manner, with the loop linking the two α-helices in the intermembrane space and their N- and C-terminal regions in the mitochondrial matrix (*SI Appendix*, Figs. S2 and S17) (27), opposite to the membrane orientation of the c-subunit itself. Both TMEM70 and TMEM242 have membrane-extrinsic C-terminal domains, the one in TMEM70 being much more extensive. The possible significance of this feature is discussed below.

**Fig. 8.**
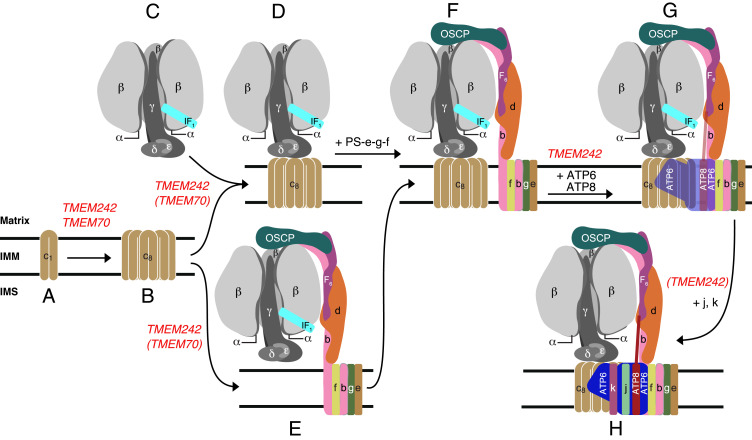
Participation of TMEM70 and TMEM242 in the assembly of c_8_-rings and their incorporation into human ATP synthase. The points where TMEM70 and TMEM242 participate are shown in red, with the parentheses indicating that their participation is suggested by the experimental data, but not proved. (*A* and *B*) Eight monomeric c-subunits are assembled into c_8_-rings. Both TMEM242 and TMEM70 participate in the formation of the membrane bound monomeric c-subunits and in the formation of c_8_-rings. (*C*–*F*) The c_8_-ring becomes associated with the separately assembled F_1_-module (*C*) to form (*D*) the F_1_-c_8_ intermediate (29). In a separate alternate pathway, the c_8_-ring is incorporated into the F_1_-PS-e-f-g vestigial complex (*E*) observed in HAP1-Δc cells (4, 10) to form a key intermediate (*F*) observed in ρ^0^ cells (4). TMEM242, and possibly TMEM70 also, participate in the incorporation of the c_8_-ring into vestigial complexes (*D* and *E*). Key intermediate *F* can be formed also from *D* by the addition of first, the PS, and then subunits e and g together, and finally subunit f. No assembly factors are known to participate in the conversion of *D* to *F* (11). The key intermediate (*F*) provides the template for the incorporation of the two mitochondrially encoded subunits, ATP6 and ATP8, which is supported by TMEM242 (*G*). The subsequent addition of subunit j stabilizes their incorporation (10). The addition of subunit k completes the monomeric enzyme (*H*). The incorporation of both subunits j and k require TMEM242. It is not known when the monomeric complexes associate into the higher oligomeric rows of back-to-face dimers observed along the edges of the cristae. The interface between monomers in back-to-face dimers involves interactions between j-subunits (23). The noncovalent cross-links between monomers in adjacent dimers across the dimer–dimer interface probably involve both g- and k-subunits (41). It is possible these cross-links form from monomeric or dimeric complexes, or both.

One striking feature of TMEM70 is that, although it influences the assembly of the ATP synthase, nonetheless the enzyme is assembled in its absence, albeit at lower levels than in its presence (Figs. 4 and 5) (18, 22). A similar feature is associated with TMEM242. In addition, TMEM70 and TMEM242 interact selectively with the c-subunit from among the 18 types of subunits present in the ATP synthase, both of them forming high molecular mass complexes with the c-subunit in the range of 60 to 150 kDa. TMEM70 was also observed in much larger complexes that may either relate to the oligomeric scaffold proposed to aid c_8_-ring assembly (27, 28) or, given the tendency of the c-subunit to aggregate in vitro, they could similarly be nonphysiological experimental artifacts. In contrast to TMEM70, TMEM242 has a wider influence as the levels of subunits ATP6, ATP8, j, and k in the vestigial complexes are diminished further in its absence, similar to the effect of removing subunit c (4). These observations suggest that TMEM70 and TMEM242 have similar and overlapping functions in the assembly of c_8_-rings, but that TMEM242 additionally influences the incorporation of subunits ATP6, ATP8, j, and k (Fig. 8). Subunits ATP6 and ATP8 are added after the incorporation of the c_8_-ring into the F_1_-PS-c_8_-e-f-g subcomplex, and the monomeric complex is completed by the addition of first, subunit j, which stabilizes the binding of ATP6 and ATP8, and finally subunit k (10) (Fig. 8). TMEM242, appears to be required for these terminal steps, whereas TMEM70 does not.

Another similarity between the effects of deletion of TMEM70 and TMEM242 is that in both instances the level of complex I is diminished, as well as that of the ATP synthase. In addition, removal of TMEM242, but not of TMEM70, reduces the levels of respiratory complexes III and IV (Fig. 4). Reduced levels of complexes I, III, and IV accompany disablement of ATP synthase by deletion of specific subunits (4, 10, 11, 29, 30). Removal of TMEM70 and TMEM242 together has a slightly greater impact on complexes III and IV to the removal of TMEM242 alone, but the reduction in the level of complex I was even more pronounced (Fig. 4 and *SI Appendix*, Fig. S7*C*). These effects on complex I and ATP synthase suggest that common features of TMEM70 and TMEM242 operate in the assembly processes of both enzyme complexes, and the association of TMEM70 and TMEM242 with the MCIA complex implies their connection in some unknown way with the assembly of complex I. MCIA is required for the assembly of the ND2 domain of the membrane intrinsic Pp-b module (proton pumping proximal-b module) of complex I (31), but TMEM70 has been proposed to be part of the P_D_-a module (proton pumping distal-a module) that becomes joined to the ND2 domain during the assembly process (32). Two subunits of complex I, NDUFB10 and FB11, components of the P_D_-a module, were more enriched with TMEM70-t than with TMEM242-t. Therefore, TMEM70-t appears to be associated more extensively with the membrane domain of complex I than TMEM242-t. The MCIA complex has six or seven protein components (33). The core of the complex is provided by ACAD9, ECSIT, and NDUFAF1. ACAD9 is a homo-dimer with each monomer in the apo-state, lacking the bound flavin molecule required for its alternative role in β-oxidation (34). Another component, TMEM126B (24), is thought to engage transiently with the core of MCIA and to anchor it to the IMM, but it is not the sole anchor (33). The loss of TMEM126B arrests assembly of complex I (24, 33, 35). TMEM186 and COA1, also found associated with MCIA in the IMM (32), have been ascribed roles in the incorporation of two of the six subunits of complex I that are encoded in the mitochondrial genome (33, 36); TMEM186 associates with newly translated ND3 of the ND2 module in the membrane arm of complex I; COA1 engages with a complex involving the mitochondrial ribosome during the translation of the ND2 subunit. A seventh protein, TIMMDC1, has also been proposed to engage transiently with the core complex in its role in uniting the Q-module (quinone binding module) with the Pp-module (35, 37). TIMMDC1 is a member of the TIM17-TIM22-TIM23 family involved in transport of proteins across the IMM (35, 37).

Thus, on the basis of current knowledge, two possible explanations concerning the roles of TMEM70 and TMEM242 can be considered. First, both TMEM70 and TMEM242 promote the integration of subunit c into the IMM and the formation of c_8_-rings, possibly by providing scaffolds to assist in this process (28), followed by association of the c_8_-rings with the F_1_-domain. The C-terminal region of yeast Mrx15, the ortholog of TMEM70 (22), interacts with the large subunit of the mitochondrial ribosome (38), suggesting that TMEM70 may also be involved in the translation and membrane insertion of ATP6 and ATP8, with TMEM242 assisting the insertion of mitochondrially encoded subunits ATP6 and ATP8. Just as MCIA is connected with the incorporation of mitochondrially encoded subunits into complex I (33), MCIA together with TMEM70 and/or TMEM242 could participate in the incorporation of ATP6 and ATP8 into ATP synthase in the latter stages of its assembly. However, the direct involvement of MCIA in the assembly of ATP synthase is not supported by the observations that the level of ATP synthase was not reduced by the deletion of ACAD9, ECSIT, or NDUFAF1 (33). Thus, it is possible that the association of TMEM70 and TMEM242 with MCIA is required solely for the assembly of the membrane arm of complex I. A second possible explanation of the association of TMEM70 and TMEM242 with MCIA is that they participate in a negative regulatory mechanism connecting the levels of complex I and ATP synthase. According to this proposal, under physiological conditions when ATP demand is high, the mitochondria would respond by increasing the level of ATP synthase, with TMEM70 and TMEM242 both involved in the assembly of the c_8_-ring and not associated with MCIA, freeing MCIA to participate in the assembly of complex I. When the demand for ATP synthase and the levels of nascent subunit c are reduced, TMEM70 and TMEM242 would sequester MCIA and thereby reduce the assembly of complex I. Thus, in view of these uncertainties, the association of TMEM70 and TMEM242 with MCIA and their roles in the assembly of ATP synthase and complex I warrant further investigation.

## Materials and Methods

HAP1-WT and clonal HAP1-ΔTMEM70 cells were purchased from Horizon Discovery. Flp-In T-REx HEK293-∆δ cells, and Flp-In T-REx HEK293 cells (known as “parental cells”) that have been modified for stable expression of METTL12 with tandem C-terminal Strep II and FLAG tags (METTL12-t), have been described previously (11, 39). Human *TMEM242* in HAP1-WT and HAP1-ΔTMEM70 cells, and *TMEM70* and *TMEM242* in HEK293-∆δ cells were disrupted by CRISPR-Cas9 (40), leading to HAP1-ΔTMEM242, HAP1-ΔTMEM70.ΔTMEM242, HEK293-Δδ.ΔTMEM70, HEK293-Δδ.ΔTMEM242, and HEK293-Δδ.ΔTMEM70.ΔTMEM242 cells. Plasmids produced from pcDNA5/FRT/TO and encoding tagged proteins c-t, g-t, TMEM70-t, or TMEM242-t were cotransfected individually together with plasmid pOG44 into Flp-In T-REx HEK293 cells or derived HEK293-Δδ cells, and stably transformed cells expressing the tagged proteins were selected. Similarly, Flp-In T-REx HeLa cells were generated that expressed TMEM242-Nt. Cell proliferation was monitored with an Incucyte HD instrument (Essen Bioscience), and the oxygen consumption of cells was measured in a Seahorse XF^e^24 analyzer (Agilent Technologies). The oligomeric state of ATP synthase and vestigial complexes was examined by CN-PAGE and Western blotting. Tagged and associated proteins, and ATP synthase and vestigial complexes, were purified from mitochondria or mitoplasts, and analyzed by SDS/PAGE with Coomassie blue or silver staining, and immunodetection of ATP synthase subunits, and by quantitative MS of trypsin and chymotrypsin digests. For quantitative MS analyses, proteins were subject to stable isotope labeling in cell culture (SILAC). For details, see *SI Appendix*.

## Supplementary Material

Supplementary File

Supplementary File

Supplementary File

Supplementary File

Supplementary File

Supplementary File

Supplementary File

Supplementary File

Supplementary File

Supplementary File

Supplementary File

Supplementary File

Supplementary File

Supplementary File

Supplementary File

Supplementary File

Supplementary File

Supplementary File

Supplementary File

Supplementary File

Supplementary File

Supplementary File

Supplementary File

Supplementary File

Supplementary File

Supplementary File

## Data Availability

All study data are included in the article and supporting information.
