# Supplementary material for: TMEM70 and TMEM242 help to assemble the rotor ring of human ATP synthase and interact with assembly factors for complex I
Source: Proc Natl Acad Sci U S A. 2021 Mar 22;118(13):e2100558118. doi: 10.1073/pnas.2100558118 (PMC8020751; doi:10.1073/pnas.2100558118)
Supplement: Supplementary File [file pnas.2100558118.sapp.pdf]

PNAS
www.pnas.org

Supplementary Information for

TMEM70 and TMEM242 help to assemble the rotor ring of human ATP synthase and interact with assembly factors for complex I

Joe Carroll, Jiuya He, Shujing Ding, Ian M. Fearnley and John E. Walker
The Medical Research Council Mitochondrial Biology Unit, University of Cambridge, Cambridge Biomedical Campus, Hills Road, Cambridge CB2 0XY, United Kingdom

Corresponding author: John E. Walker

Email: walker@mrc-mbu.cam.ac.uk

This PDF file includes:

Supplementary Materials and Methods
Figures S1 to S17
Tables S1 to S3
Legends for Datasets S1 to S25
SI References

Supplementary Materials and Methods

General Methods. Cell protein concentrations were determined by either the bicinchoninic acid assay (Thermo Fisher Scientific) or the detergent compatible protein assay (BioRad). Mitoplasts were prepared from cells with digitonin, as described before (1, 2). Extracts of mitoplasts made with DDM (1%, w/v) were fractionated by SDS-PAGE, and subunits of ATP synthase and citrate synthase were detected by Western blotting. The oligomeric states of ATP synthase and vestigial complexes in digitonin extracts of mitoplasts were examined by BN-PAGE or CN-PAGE (3, 4), and Western blotting. Samples of mitoplasts were re-suspended to *ca.* 5 mg/mL in Native PAGE sample buffer (Thermo Fisher Scientific) containing digitonin (6-12 g/g protein), kept at 4°C for 15 min, and then centrifuged (10,500 x *g*, 20 min, 4°C). The supernatants were treated with benzonase (Merck Millipore) at room temperature, centrifuged again, and soluble complexes fractionated at 4°C in 3-12% acrylamide gradient Bis-Tris gels (Thermo Fisher Scientific) by CN-PAGE, or BN-PAGE according to the manufacturer's instructions for "Western blotting". For CN-PAGE the cathode running buffer contained 0.05% (w/v) sodium deoxycholate plus 0.005% (w/v) DDM. Complexes were transferred from the gels to polyvinylidene fluoride membranes, and the membranes were probed with subunit specific antibodies (see Table S3). ATP synthase was purified from digitonin solubilized mitoplasts with an immuno-capture resin (Abcam) as described before (5). Proteins in SILAC labelled mitoplast samples for quantitative mass spectrometric (MS) analysis were extracted with either digitonin (detergent:protein, 10:1 or 12:1, g:g) or DDM (detergent:protein, 5:1, g:g), reduced and alkylated in gel sample buffer, fractionated by SDS-PAGE and stained with Coomassie blue R250 dye. Stained gel sections were excised and proteins digested in-gel with trypsin or chymotrypsin (6). SILAC labelled and affinity purified samples of ATP synthase were ethanol precipitated at -20ºC for 18 h with 20 vol. cold ethanol, centrifuged, and the pellet was digested in 50 mM ammonium bicarbonate for 18 h, with either trypsin at 37ºC or chymotrypsin at 30ºC. The procedure for the affinity purification of tagged proteins from human mitochondria and mitoplasts has been described before (1, 7), except that proteins were extracted with digitonin (see above) in the presence of a buffer consisting of 50 mM Tris-HCl, pH 8.0, 150 mM NaCl, 1-palmitoyl-2-oleoyl-*sn*-glycero-3-phosphocholine (0.09 mg/mL), 1-palmitoyl-2-oleoyl-*sn*-glycero-3-phosphoethanolamine (0.03 mg/mL), 1-palmitoyl-2-oleoyl-*sn*-glycero-3-phosphoglycerol (0.03 mg/mL), 1x cOmplete EDTA-free protease inhibitor (Roche) and 2 mM dithiothreitol. After loading the sample in this buffer, the column was washed with the buffer plus 0.05% w/v digitonin, and eluted with the buffer where the lipid was replaced by 10 mM desthiobiotin.

Cell Culture. HAP1-WT and HAP1-ΔTMEM70 clonal cells (catalog number HZGHC003615c010) were obtained from Horizon Discovery. They and other HAP1 clonal cells were cultured in Iscove's modified Dulbecco's medium under standard conditions (5). Flp-In^{TM} T-REx^{TM} HEK293 cells (Thermo Fisher Scientific) and derivative clonal cells were cultured in Dulbecco's Modified Eagle Medium (DMEM) containing zeocin (100 µg/mL) and blasticidin (10 µg/mL). For the stable expression of proteins, Flp-In^{TM} T-REx^{TM} HEK293 cells and derivatives were grown in DMEM, containing tetracycline free fetal bovine serum (10%), hygromycin (100 µg/mL) and blasticidin (10 µg/mL). Flp-In^{TM} T-REx^{TM} HeLa cells were grown under the same conditions except that the concentration of blasticidin was 2 µg/mL. Stable isotope labelling of proteins with amino acids in cell culture (SILAC) (8) of HAP1 and HEK293 cells, and of clonal cells containing disrupted genes, was carried out as described before (1, 5). HEK293 parental cells and two clonal cells were subjected also to a three-way SILAC labelling procedure. Cells were grown at 37°C with an atmosphere of 5% CO_2 in DMEM media minus L-arginine and L-lysine (Thermo Scientific) supplemented with dialyzed fetal bovine serum (10% v/v), penicillin (100 U/mL), streptomycin (0.1 mg/mL), proline (200 mg/L) and 20 mM HEPES, pH 7.4. L-arginine (0.398 mM) and L-lysine (0.798 mM) were added in the following combinations: light labelled ($^{12}C_6{}^{14}N_4$-Arg, $^{12}C_6{}^{14}N_2$-Lys); medium labelled ($^{13}C_6{}^{14}N_4$-Arg, $^{2}D_4$-Lys); heavy labelled ($^{13}C_6{}^{15}N_4$-Arg, $^{13}C_6{}^{15}N_2$-Lys). Cell proliferation was measured as percent confluence with an Incucyte HD instrument (Essen Bioscience). Rates of oxygen consumption (OCR) and extracellular acidification (ECAR) were measured in a Seahorse XF^{e}24 analyzer (Agilent Technologies), as described previously (5). The cellular protein content was determined with the sulforhodamine B assay (9). OCR and ECAR values were normalized to the number of cells with a standard linear calibration of cell number versus the absorbance of sulforhodamine B.

Disruption of Genes. The structures of *TMEM70* and *TMEM242* are shown in Fig. S3. Exons I in *TMEM70* in HEK293-Δδ Flp-In^{TM} T-REx^{TM} cells and in *TMEM242* in HAP1-WT cells were disrupted by CRISPR-Cas9 (10) with the pairs of gRNAs given in Table S1. *TMEM242* was disrupted also in HAP1-ΔTMEM70 cells and in HEK293-ΔδΔTMEM70 cells with the same gRNAs. HEK293-ΔTMEM70 clonal

cells were obtained by screening clones from single cells with an antibody against TMEM70. As no antibody against TMEM242 was available for identifying HAP1-ΔTMEM242 cells by a similar approach, clonal cells with deletions in *TMEM242* were identified by screening the targeted regions of the genes by amplifying them by PCR and by sequencing the amplified fragments. The three disrupted alleles of *TMEM242* in HEK293 cells were characterized in similar way.

***TMEM70*.** The 32 nucleotide deletion in exon I of *TMEM70* in HAP1-ΔTMEM70 cells resulted in the possibility of a transcript encoding a C-terminally truncated 75 amino acid protein in which the C-terminal 28 amino acids do not correspond to the sequence of TMEM70 (*SI Appendix* Fig. S4*A* and *B*). In HEK293-Δδ.ΔTMEM70 cells a 78 nucleotide deletion in *TMEM70* exon I removed the translational initiation codon (*SI Appendix* Fig. S10*A*), and there was no potential second initiation codon in exon I downstream of the deletion. Neither cell line expressed TMEM70 (*SI Appendix* Fig. S5).

***TMEM242*.** A 113 nucleotide deletion in HAP1-ΔTMEM242 cells removed the majority of exon I of *TMEM242*, including the translation initiation codon, and 10 nucleotides from the 5' upstream sequence (*SI Appendix* Fig. S4*C*). There is no additional initiation codon in exon I, and no significant level of an *TMEM242* transcript was observed (*SI Appendix* Fig. S5*B*). HAP1-ΔTMEM70 cells provided the parent for the HAP1-ΔTMEM70.ΔTMEM242 clone, where a 120 nucleotide deletion in *TMEM242* removed most of exon I, including the translation initiation codon and 14 nucleotides of the upstream 5' sequence (*SI Appendix* Fig. S4*D*), preventing any significant level of transcription (*SI Appendix* Fig. S5*B*). In the polyploid HEK293-Δδ.ΔTMEM242 cells, three different modifications of *TMEM242* were identified (*SI Appendix* Fig. S10*B*-*D*). In two instances, each individual gRNA produced a different nucleotide deletion (*SI Appendix* Fig. S10*B* and *C*) where 2 or 30 nucleotides, respectively, preceding the translational initiation codon were deleted in the 5' upstream region before exon I plus the first base of exon I. Additional deletions of 1 or 7 nucleotides at the 3' end of exon I, each created a frame-shift and a premature translation termination codon, resulting in a potential transcript encoding a 42 or 40 amino acid polypeptide, with 13 and 12 mismatched amino acids, respectively, in the C-terminal region (*SI Appendix* Fig. S10*B* and *C*). In the third instance, a 120 nucleotide deletion removed 17 nucleotides from the upstream 5' sequence of *TMEM242* and the majority of exon I, including the translational initiation codon (*SI Appendix* Fig. S10*D*). Transcripts from the HEK293-Δδ.ΔTMEM242 cells (*SI Appendix* Fig. S5*B*) were sequenced across the exon I-II boundary and five out of thirteen of them corresponded to one of the gene deletions (Fig. S10*C*). The remainder corresponded to a deletion across the exon I-II boundary. No sequence was obtained for the second transcript (Fig. S10*B*), presumably because it is rare. The HEK293-Δδ.ΔTMEM70.ΔTMEM242 clone, derived from HEK293-Δδ.ΔTMEM70 cells, arose from a 115 nucleotide deletion in *TMEM242* including 12 nucleotides from the upstream 5' sequence, plus the majority of exon I including the translational initiation codon (*SI Appendix* Fig. S10*E*). No significant level of transcription was observed (*SI Appendix* Fig. S5*B*).

Transcription of *TMEM242*, *ATP5MC1*, *ATP5MC2* and *ATP5MC3*. The levels of transcripts for TMEM242, subunit c from *ATP5MC1*, *ATP5MC2* and *ATP5MC3*, relative to the level of the transcript for β-actin, were determined in the following cells: HAP1-WT, HAP1-ΔTMEM242, HAP1-ΔTMEM70.ΔTMEM242, HEK293-Δδ, HEK-Δδ.ΔTMEM242 and HEK-Δδ.ΔTMEM70.ΔTMEM242. The transcripts were amplified by qPCR in an ABI QuantStudio™ 3 Real-Time PCR instrument, with specific TaqMan gene expression assays and reagents (Thermo Fisher Scientific). The relative levels of transcripts were calculated in Excel by the ΔΔCt (cycle threshold) method. The mixtures of primers and probes for estimating the levels of transcripts from *TMEM242*, *ATP5MC1*, *ATP5MC2* and *ATP5MC3* were Hs00218395_m1, Hs00829069_s1, Hs01096582_m1 and Hs00266086_m1, with the TaqMan MGB (minor groove binder) probes, with a 5' 6-FAM (6-carboxyfluorescein) reporter dye and a non-fluorescent quencher at their 3'-extremities. The probe for *TMEM242* probe hybridizes at the boundary of exons 3 and 4. The forward and reverse primers for β-actin were 5'-CCTGGCACCCAGCACAAT-3', and 5'-GCCGATCCACACGGAGTACT-3', respectively; the probe was 5'-[6-FAM] ATCAAGATCATTGCTCCTCCTGAGCGC [TAM]-3' where TAM is 5-carboxy-tetramethylrhodamine.

Sources of Antibodies. See Table S3, except for those against subunits α, β, γ, b, d, c, g, j and ATP8 of ATP synthase, Strep II, citrate synthase, TIM23, SDHA, NDUFS2, UQCRC1 and COX4, which have been described before (11–13).

Expression of Tagged Mitochondrial Proteins. Two expression plasmids were prepared with the coding sequences for subunit c-t encoded in *ATP5MC3* and g-t in *ATP5MG*, cloned between the *Hin*d III and *Xho* I sites of plasmid pcDNA5™/FLP/TO (Thermo Fisher Scientific). The coding sequences for

TMEM70-t, TMEM242-t, or TMEM242-Nt, were introduced into the same plasmid between its *Bam*H I and *Xho* I sites. The proteins were expressed in HEK293 Flp-In^{TM} T-Rex^{TM}, HEK293-Δδ Flp-In^{TM} T-Rex^{TM} cells, and HeLa Flp-In^{TM} T-Rex^{TM} cells. Expression of proteins was induced for 72-96 h with doxycycline (20 ng/mL). TMEM242-t and TMEM242-Nt were each expressed transiently in HeLa cells. After 48 h of growth, the cells were fixed with formaldehyde, permeabilized and examined by confocal microscopy.

Confocal Microscopy. TMEM242-t and TMEM242-Nt were detected in transiently transfected HeLa cells with a mouse anti-FLAG primary antibody (Table S3) and a goat anti-mouse secondary antibody labelled with the fluorescent dye, Alex-Fluro®488. TOM20 was detected with rabbit polyclonal anti-TOM20 primary antibody (Table S3) and the Alex-Fluro®647 fluorescently labelled goat anti-rabbit secondary antibody. Fluorescently labelled cells in the presence of ProLong Gold Antifade Mountant (Thermo Fisher Scientific) plus the nuclear stain, 4′,6-diamidino-2-phenylindole (Invitrogen) were imaged with a Zeiss LSM 880 confocal microscope equipped with LSM 880 software. Images were acquired with a Zeiss 63 x oil immersion objective, set at zoom 2.4. Images were edited in Affinity designer.

Mitochondrial Membrane Association of TMEM242. TMEM242-t and TMEM242-Nt were expressed stably in Flp-In^{TM} T-REx^{TM} HEK293 and Flp-In^{TM} T-REx^{TM} HeLa cells, respectively. Cells were disrupted by hypotonic lysis and homogenization, and fractionated by differential centrifugation. Purified mitochondria were resuspended at 4°C in 0.1 M Na_2CO_3, pH 11.5, to a protein concentration of 0.1 mg/mL and kept at 4°C for 30 min. The suspension was centrifuged (4°C; 233,000xg; 1 h), and the supernatant was retained. The pellet consisting of mitochondrial membranes was rinsed at 4°C with milli-Q water, resuspended in 0.1 M Na_2CO_3 at 4°C, and then extracted with increasing concentrations of deoxycholate for 30 min. The residual extracted pellet was recentrifuged. The various pellets and supernatants were fractionated by SDS-PAGE and specific proteins were detected with antibodies (Table S3).

Membrane Topology of TMEM242. Purified mitochondria were resuspended in 1 x MSH buffer and the agitated suspension was digested at 25°C for 30 min with various concentrations of trypsin. Digestion was terminated by the addition of a five-fold molar excess of bovine pancreatic trypsin inhibitor (Sigma) and cOmplete^{TM} proteinase inhibitor cocktail (Roche). Mitochondria that had been solubilized with 1% (w/v) n-dodecyl-β-D-maltoside (DDM) were digested with trypsin in a similar way.

Protein Quantitation. Relative quantitation of proteins in SILAC samples (8) was performed as described before (13) by analysis of tryptic and chymotryptic peptide digests of proteins. Peptide mass data were analyzed with MaxQuant version 1.6.5.0 or 1.6.10.43, and the integrated Andromeda search engine (14, 15). The procedures for processing and analysis of the data have been described previously (13). The identification of subunit c in the MS analyses of SILAC samples depends principally on the observation of two chymotryptic peptides, residues 1-8 and 37-47. To a lesser extent, larger partial products containing these peptides are observed also. Peptide 37-47 contains trimethylated Lys-43, and therefore the data interrogation scheme includes this modification (13). Subunit c has only two potential tryptic cleavage sites and none of the tryptic peptides is recovered consistently from the liquid chromatography (LC) procedure incorporated in the LC-MS analyses, although tryptic peptide 8-38 is observed sometimes.

Supplementary Figures

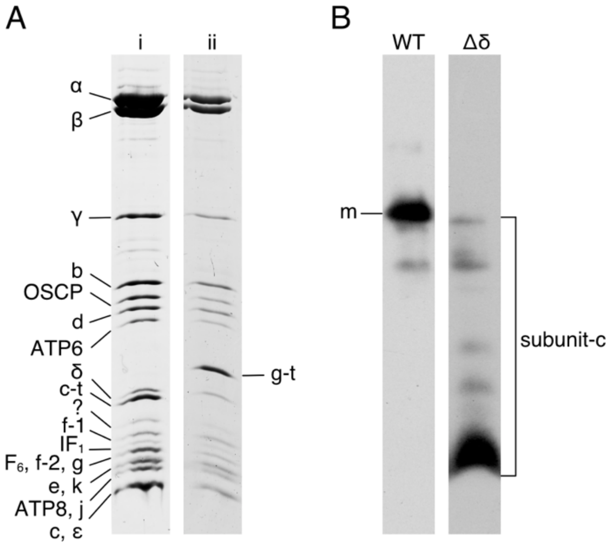

Fig. S1. Incorporation of tagged subunits c and g into human ATP synthase. (*A*) Analysis by SDS-PAGE of ATP synthases containing in: lane i, subunit c-t and lane ii, subunit g-t. Proteins were detected with Coomassie blue dye. The locations of subunits are indicated on the left. Expression of subunits c-t and g-t was induced in HEK293 Flp-In^{TM} T-REx^{TM} cells for 3 days with doxycycline (20 ng/mL). Digitonin extracts (detergent:protein, 10:1, g:g) of mitochondria were applied to a Strep II column, and ATP synthase was released with D-desthiobiotin. (*B*) Analysis by BN-PAGE of digitonin extracts of mitoplasts from HEK293-WT and HEK293-Δδ cells. The bands were detected with an antibody against subunit c; m, ATP synthase monomer.

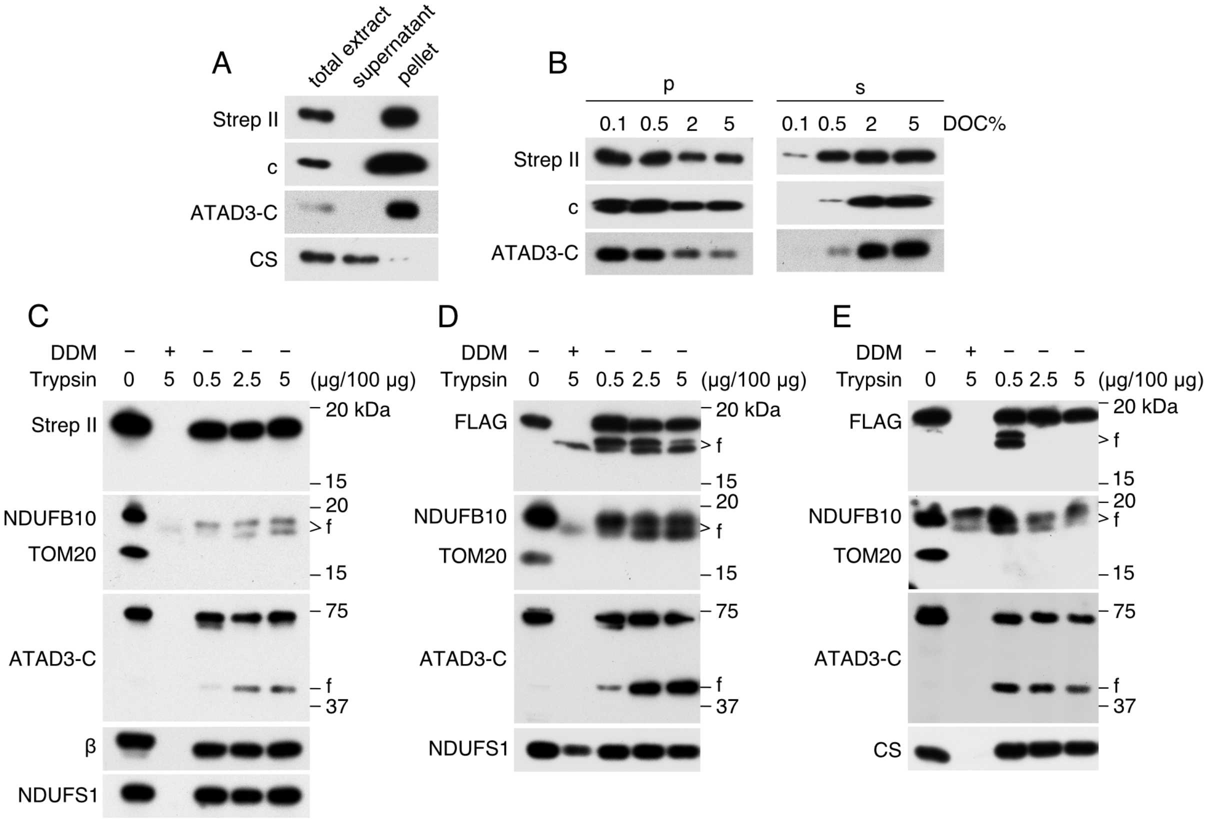

Fig. S2. Membrane topology of TMEM242. Analyses by SDS-PAGE and immunodetection of proteins indicated on the left of each panel in the following samples: (*A*) extract made at 4ºC for 30 min with 0.1 M Na_2CO_3, pH 11.5, of mitochondria from Flp-In™ T-REx™ HEK293 cells expressing TMEM242 with C-terminal tandem Strep II and FLAG tags; the extract was centrifuged (233,000 x *g*, 60 min, 4ºC) and the supernatant and pellet fractions were collected; (*B*) pellet from (*A*) extracted at 4ºC for 30 min with increasing concentrations of deoxycholate (DOC, % w/v), and then centrifuged as above; p, pellet; s, supernatant; (*C-E*), trypsinized samples of mitochondria from the following cells; (*C*) Flp-In™ T-REx™ HEK293 cells expressing TMEM242 with tandem C-terminal Strep II and FLAG tags following induction of expression for 96 h with doxycycline (20 ng/mL); (*D* and *E*) HeLa cells induced to express TMEM242 with an N-terminal FLAG tag for 96 h with doxycycline, 20 ng/mL in (*D*), and 2 ng/mL in (*E*). The mitochondria were digested for 30 min at 25ºC with trypsin (at concentrations in µg/100 µg protein indicated above the panels); above the tracks, - and + indicate, respectively, samples that were trypsinized directly, and those that were first lysed with 1% (w/v) DDM and then trypsinized. Digestion was terminated by the addition of a 5-fold molar excess of bovine pancreatic trypsin inhibitor. The sizes of protein markers are shown on the right. Proteins were detected with antibodies against the following: Strep II or FLAG tags; amino acids 264-617 in the C-terminal region of ATAD3, located in the mitochondrial matrix (16); the mitochondrial outer membrane protein TOM20; the NDUFB10 subunit of complex I which is exposed in the IMS; the NDUFS1 subunit of complex I and the β-subunit of ATP synthase, both of which are located on the matrix side of the IMM; CS, citrate synthase found in the matrix. On the right, the letter f denotes protein fragments that are protected from trypsinization by the IMM.

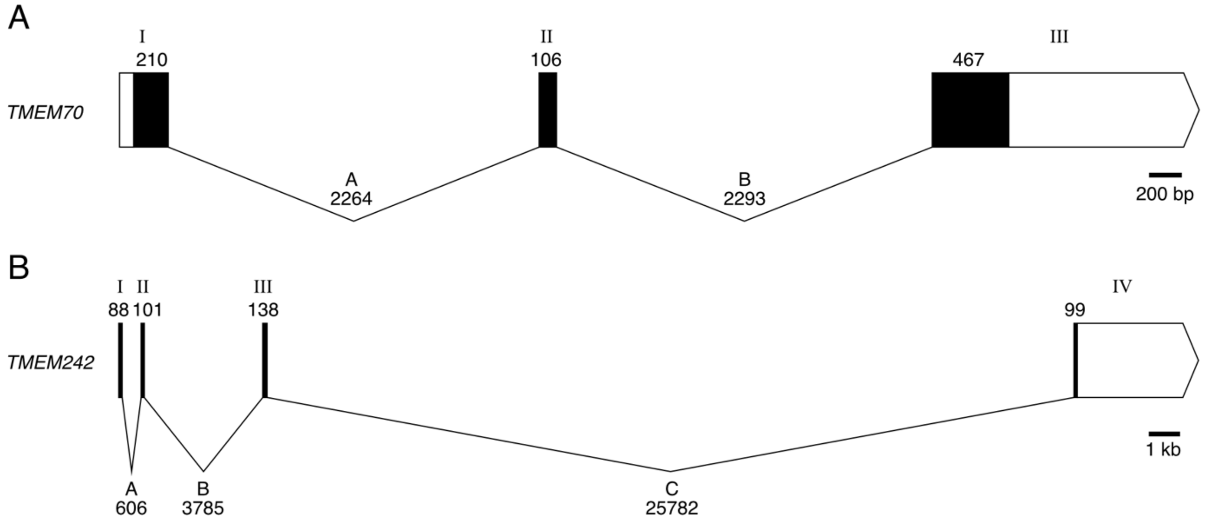

Fig. S3. Structures of human *TMEM70* and *TMEM242*. (*A*), *TMEM70* on chromosome 8; (*B*), *TMEM242* on chromosome 6. Boxes labelled with Roman numerals represent exons, and black and non-filled areas indicate protein coding and non-coding regions, respectively. The intervening continuous lines denote introns. The sizes of introns and coding regions of exons are given in base pairs (bp). The exon-intron information was obtained from http://www.ensembl.org and transcript ID reference ENST00000312184.6 for *TMEM70* and ENST0000400788.9 for *TMEM242*. The gene structures were drawn with the Exon-Intron graphic maker (http://wormweb.org/exonintron).

```
A
                             →
TMEM70      CGCTCGTCACCCGCGTGATGCTGTTTCTGGCGTTGGGCAGCCCGTGGGCGGTCGAACTGCCTCTCTGCGG
ΔTMEM70     CGCTCGTCACCCGCGTGATGCTGTTTCTGGCGTTGGGCAGCCCGTGGGCGGTCGAACTGCCTCTCTGCGG

TMEM70      AAGGAGGACTGCATTGTGTGCGGCCGCCGCGCTCCGAGGTCCCCGGGCCTCTGTCTCCCGGGCGTCCTCC
ΔTMEM70     AAGGAGGACTGCATTGTGTGCGGCCGCCGCGCTCCGAGGTCCCCGGGCCTCTGTCTCCCGGGCGTCCTCC
                                      ____________________^^^
TMEM70      AGCAGCGGGCCTTCGGGGCCGGTAGCCGGCTGGAGTACGGGGCCTTCGGGAGCCGCGCGCCTTCTCCGGC
ΔTMEM70     AGCAGCGGGCCTTCGGGG--------------------------------AGCCGCGCGCCTTCTCCGGC
```

```
B
WT         1 MLFLALGSPWAVELPLCGRRTALCAAAALRGPRASVSRASSSSGPSGPVAGWSTGPSGAARLLRRPGRAQ  70
ΔTMEM70    1 MLFLALGSPWAVELPLCGRRTALCAAAALRGPRASVSRASSSSGPSGSRAPSPASGSSADPCLLGRICSI  70
                                                            ** ****** * *** *******

WT        71 IPVYWEGYVRFLNTPSDKSEDGRLIYTGNMARAVFGVKCFSYSTSLIGLTFLPYIFTQNNAISESVPLPI 140
ΔTMEM70   71 LKYAI-----------------------------------------------------------------  75
             *****

WT       141 QIIFYGIMGSFTVITPVLLHFITKGYVIRLYHEATTDTYKAITYNAMLAETSTVFHQNDVKIPDAKHVFT 210
ΔTMEM70   71 ------------------------------------------------------------  75

WT       181 TFYAKTKSLLVNPVLFPNREDYIHLMGYDKEEFILYMEETSEEKRHKDDK 260
ΔTMEM70   71 --------------------------------------------------  75
```

```
C
                                             ^^^____________________
TMEM242     TTCTCCTCCGCCTGGTTCTCGCTTCCACCCGGGCCCAGTTGGGGACGGACGCACTAGAGGCGACCTAAAC
ΔTMEM242    TTCTCCTCCGCCTGGTTCTCGCTTCCACCCGGGCCCAGTT------------------------------
            →                                                                 ____
TMEM242     ATGGAGACAGCGGGCGCTGCAACTGGGCAGCCGGCCTCTGGGCTGGAGGCTCCGGGGTCCACGAATGACC
ΔTMEM242    ----------------------------------------------------------------------
            ________________^^^
TMEM242     GGCTTTTCCTGGTTAAAGGTAAGATGTGCTGTGGTGAGGTGCGGGCGTCGGGGTGAGTTAACCCCGGACC
ΔTMEM242    -------------TAAAGGTAAGATGTGCTGTGGTGAGGTGCGGGCGTCGGGGTGAGTTAACCCCGGACC
```

```
D
                                             ^^^____________________
TMEM242     TTCTCCTCCGCCTGGTTCTCGCTTCCACCCGGGCCCAGTTGGGGACGGACGCACTAGAGGCGACCTAAAC
ΔTMEM242    TTCTCCTCCGCCTGGTTCTCGCTTCCACCCGGGCCC----------------------------------
            →                                                                 ____
TMEM242     ATGGAGACAGCGGGCGCTGCAACTGGGCAGCCGGCCTCTGGGCTGGAGGCTCCGGGGTCCACGAATGACC
ΔTMEM242    ----------------------------------------------------------------------
            ________________^^^
TMEM242     GGCTTTTCCTGGTTAAAGGTAAGATGTGCTGTGGTGAGGTGCGGGCGTCGGGGTGAGTTAACCCCGGACC
ΔTMEM242    ----------------AGGTAAGATGTGCTGTGGTGAGGTGCGGGCGTCGGGGTGAGTTAACCCCGGACC
```

Fig. S4. Sequences of disrupted *TMEM70* and *TMEM242* and encoded proteins. The disruptions were performed in HAP1 cells. Carets indicate the PAM (protospacer adjacent motif) sequences, solid lines the target sequence, the arrow the translational initiation codon, and dashes deleted regions. (*A*) alignment of exons I in HAP1-WT and HAP1-ΔTMEM70 clonal cells where there is a 32 bp deletion; (*B*) changes in the protein sequence of TMEM70 in HAP1-ΔTMEM70 cells indicated by asterisks and deletions by dashes; (*C*) and (*D*), disruption of exon I of *TMEM242* in HAP1-WT and HAP1-ΔTMEM70 cells (see panel *A*), respectively, generating HAP1-ΔTMEM242 and HAP1-ΔTMEM70.ΔTMEM242 clonal cells, with the sequences of the targeted region aligned with the parent sequence. Part of the 5' upstream non-coding sequence and part of intron A are in a grey box in the parent sequence. By use of two gRNAs most of exon I, including the initiator methionine codon were removed with effects on both transcription and translation.

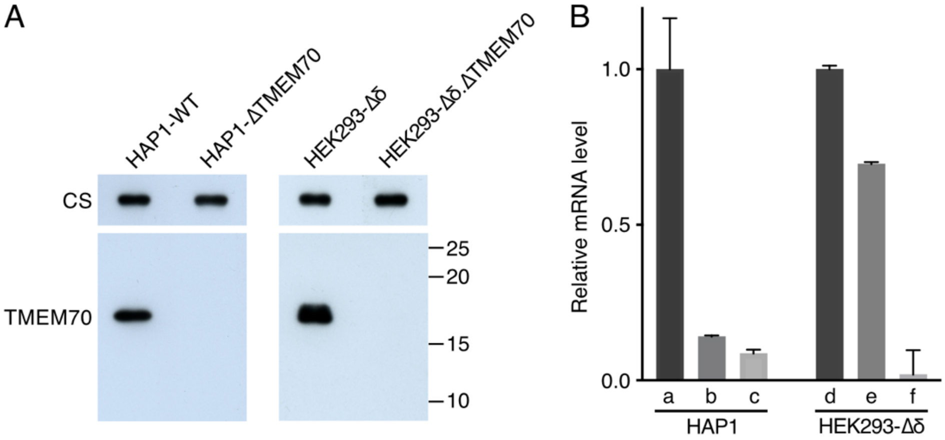

Fig. S5. Absence of TMEM70 from ∆TMEM70 cells and relative levels of transcripts from *TMEM242* in ∆TMEM242 cells. (*A*) analysis by SDS-PAGE of DDM extracts of mitoplasts from HAP1-WT, HAP1-ΔTMEM70, HEK293-∆δ and HEK293-∆δ.ΔTMEM70 cells. TMEM70 and citrate synthase (CS; loading control) were detected with an antibody. The positions of molecular weight markers (kDa) are shown on the right. (*B*) The levels of the mRNA for TMEM242 relative to that for β-actin shown as the mean plus the standard error of the mean (n=3). a, HAP1-WT cells; b, HAP1-∆TMEM242 cells; c, HAP1-∆TMEM70.∆TMEM242 cells; d, HEK293-Δδ cells; e, HEK293-∆δ.∆TMEM242 cells; f, HEK293-∆δ.∆TMEM70.∆TMEM242 cells.

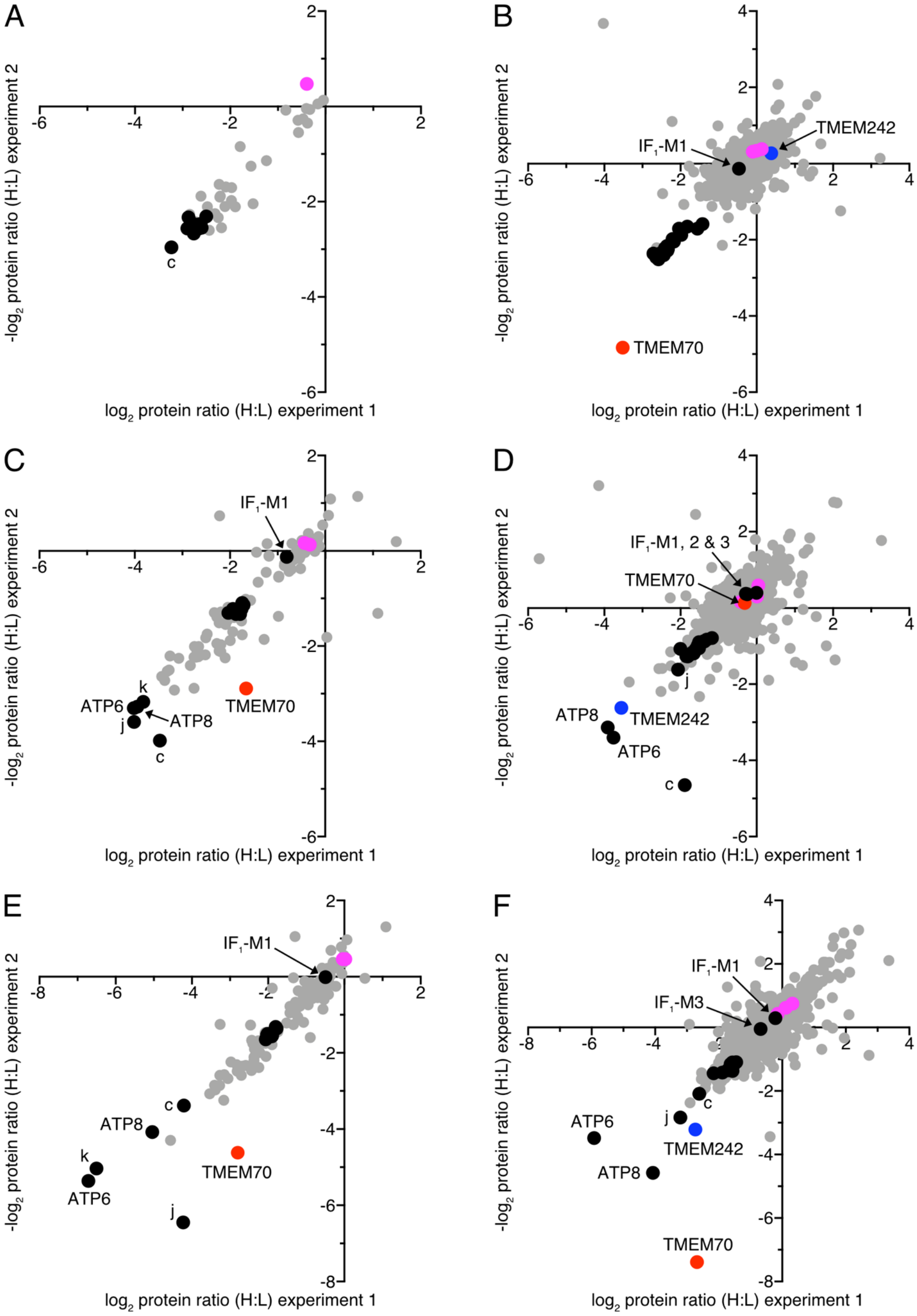

Fig. S6. Relative protein abundances in purified ATP synthase complexes and in mitoplasts from HAP1-ΔTMEM70, -ΔTMEM242 or -ΔTMEM70.ΔTMEM242 cells. ●, TMEM70; ●, TMEM242; ●, ATP synthase subunits and forms of IF_1; ●, assembly factors ATPAF1, ATPAF2 or FMC1; ●, all other proteins. (*A*, *C* and *E*) immunocaptured ATP synthase complexes, and (*B*, *D* and *F*) mitoplast proteins, from HAP1-ΔTMEM70, HAP1-ΔTMEM242 or HAP1-ΔTMEM70.ΔTMEM242 cells, respectively. Samples were prepared from a 1:1 mixture of HAP1-WT cells and HAP1-ΔTMEM70, HAP1-ΔTMEM242 or HAP1-ΔTMEM70.ΔTMEM242 cells that had been differentially SILAC-labelled. The experiments were performed twice with reciprocal SILAC labelling orientations. The protein ratio is derived from a minimum of two peptide ratios from each experiment, except for subunit c (in panel *A*, both experiments) and for ATP6 in experiment 1 in (*C*), where values are from a single peptide ratio. In (*B*), no ratio for subunit c was obtained. In (C) the TMEM70 experiment 1 ratio was re-calculated using the median peptide value, replacing the 'plateau' value from MaxQuant. The ratios for proteins obtained in both experiments are plotted as a single point on a scatter plot as the log base 2 value. In (*B*), the data point for DIS3-like exonuclease 2 (-2.49, 8.39) is outside the axes, in the upper left 'contaminant' quadrant. Protein ratios are given in *SI Appendix*, Datasets S4-S20.

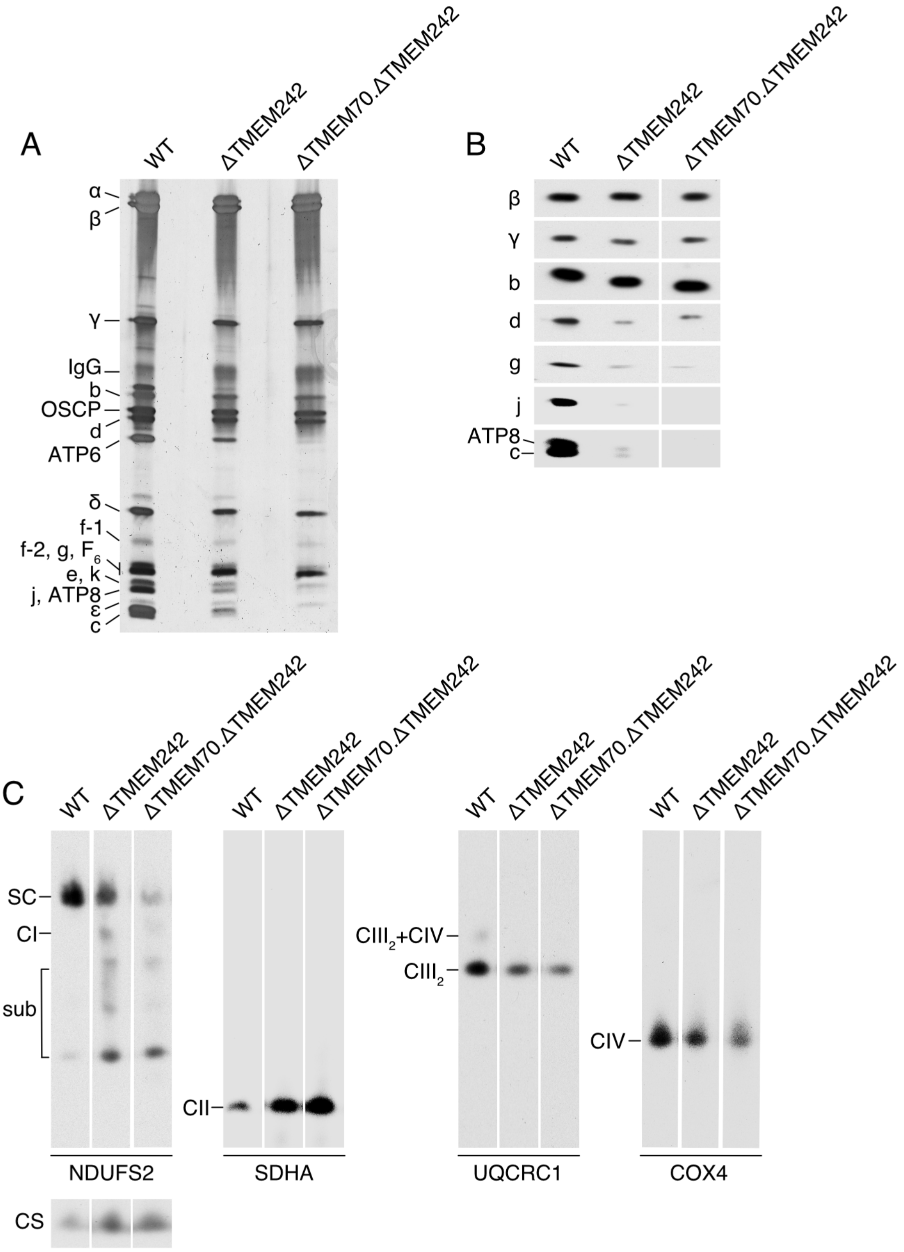

Fig. S7. Effect of deletion of TMEM242 on ATP synthase and respiratory complexes in HAP1 cells. Samples were taken from HAP1-WT, HAP1-ΔTMEM242, and HAP1-ΔTMEM70.ΔTMEM242 cells, indicated, respectively, above each panel; (*A*) and (*B*), SDS-PAGE analyses of purified ATP synthase and vestigial complexes. The samples contained approximately equivalent levels of the α- and β-subunits. In (*A*), the gel was silver-stained, and the positions of subunits are indicated on the left; in (*B*), excised bands from a duplicate SDS-PAGE analysis were immunodetected; (*C*) BN-PAGE analyses of digitonin extracts (detergent:protein, 10:1, g:g) of mitoplasts. Three times more protein was analyzed in lanes ii and iii than in lane i. Complexes I-IV were detected with the antibodies against the proteins indicated beneath the panels. The identities of complexes are shown on the left of the panels; CI, complex I; CII, complex II; $CIII_2$, complex III dimer; CIV, complex IV; $CIII_2$+CIV, complex III dimer plus complex IV; SC, supercomplex; sub, subcomplexes. In (*C*), citrate synthase (CS) provided a loading control.

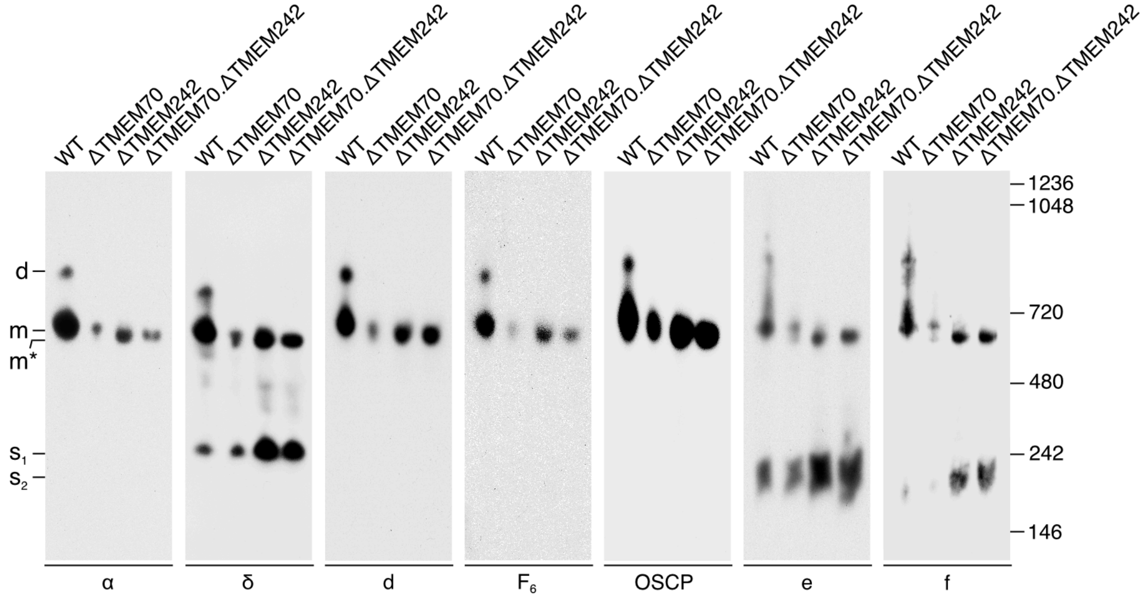

Fig. S8. Oligomeric states of ATP synthase and vestigial complexes in HAP1 cells devoid of TMEM70 and TMEM242. This Figure complements Fig. 5. In each panel, CN-PAGE analysis of digitonin extracts (detergent:protein, 10:1, g:g) of mitoplasts from HAP1-WT; HAP1-ΔTMEM70; HAP1-ΔTMEM242; and HAP1-ΔTMEM70.ΔTMEM242 cells. Below each panel is shown the specificity of the antibody for an individual subunit of ATP synthase employed to detect the complexes related to ATP synthase shown on the left; d, dimers; m, monomers; m*, the F_1-PS-e-f-g complex; s_1, incompletely characterized subcomplex containing central stalk subunits δ and ε; s_2, subcomplex of subunits b, e, f and g. The positions of molecular mass markers (kDa) are shown on the right. The total protein in the sample from HAP1-ΔTMEM70 cells was twice that from HAP1-WT cells, and those from HAP1-ΔTMEM242 and HAP1-ΔTMEM70.ΔTMEM242 cells were three times greater than HAP1-WT cells.

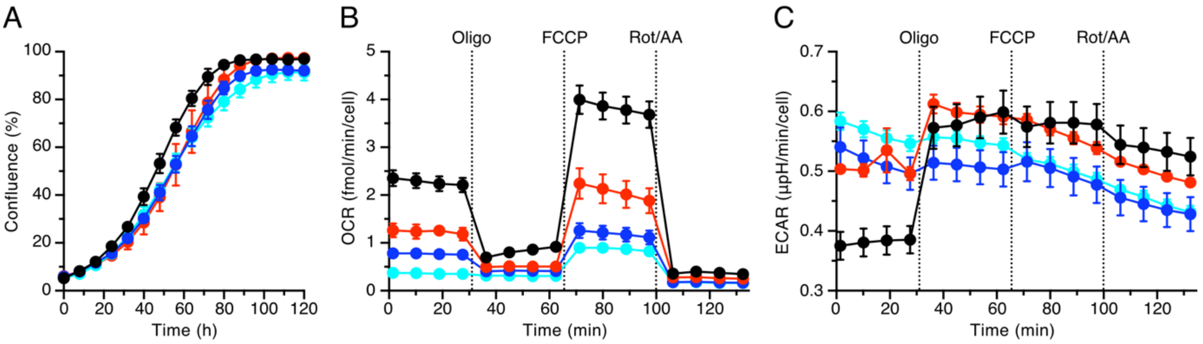

Fig. S9. Characteristics of HAP1-ΔTMEM70, HAP1-ΔTMEM242 and HAP1-ΔTMEM70.ΔTMEM242 cells. (*A*) Growth rates, (*B*) Cellular oxygen consumption rates (OCR), and (*C*) extracellular acidification rates (ECAR), of HAP1-WT cells (●), HAP1-ΔTMEM70 (●), HAP1-ΔTMEM242 cells (●), and HAP1-ΔTMEM70.ΔTMEM242 cells (●). Growth rates are the mean ± SD; n=3 wells. OCR and ECAR were measured before and after sequential additions of oligomycin (Oligo), carbonyl cyanide-4-(trifluoromethoxy)phenylhydrazone (FCCP), and a mixture of rotenone and antimycin A (Rot/AA). Data represent the mean ± SEM (n=5 wells).

```
A   TMEM70      ACTTGTGCGGCAGTCGGGTGGGAAGCCGTGTCTCGCAGTCGTGGACTCGTGCAGCTGGGG
    ΔTMEM70     ACTTGTGCGGCAGTCGGGTGGGAAGCCGTGTCTCGCA-----------------------

    TMEM70      CGTCCGCAGCCGCTCGTCACCCGCGTGATGCTGTTTCTGGCGTTGGGCAGCCCGTGGGCG
    ΔTMEM70     -------------------------------------------------------GGGCG

    TMEM70      GTCGAACTGCCTCTCTGCGG
    ΔTMEM70     GTCGAACTGCCTCTCTGCGG

B   TMEM242     AGATTGCACAGGAGGAGGCGGTAGCGGAACCTTTCTCCTCCGCCTGGTTCTCGCTTCCAC
    ΔTMEM242    AGATTGCACAGGAGGAGGCGGTAGCGGAACCTTTCTCCTCCGCCTGGTTCTCGCTTCCAC

    TMEM242     CCGGGCCCAGTTGGGGACGGACGCACTAGAGGCGACCTAAACATGGAGACAGCGGGCGCT
    ΔTMEM242    CCGGGCCCAGT--GGGACGGACGCACTAGAGGCGACCTAAACATGGAGACAGCGGGCGCT

    TMEM242     GCAACTGGGCAGCCGGCCTCTGGGCTGGAGGCTCCGGGGTCCACGAATGACCGGCTTTTC
    ΔTMEM242    GCAACTGGGCAGCCGGCCTCTGGGCTGGAGGCTCCGGGGTCCACGAATGACCGGCTTTTC

    TMEM242     CTGGTTAAAGGTAAGATGTGCTGTGGTGAGGTGCGGGCGTCGGGGTGAGTTAACCCCGGA
    ΔTMEM242    CTGG-TAAAGGTAAGATGTGCTGTGGTGAGGTGCGGGCGTCGGGGTGAGTTAACCCCGGA

    WT          1  METAGAATGQPASGLEAPGSTNDRLFLVKGGIFLGTVAAAGMLAGFITTLSLAKK  55
    ΔTMEM242    1  METAGAATGQPASGLEAPGSTNDRLFLVKVEFSLVPLLQREC-------------  42
                                               **** ********
    WT         56  KSPEWFNKGSMATAALPESGSSLALRALGWGSLYAWCGVGVISFAVWKALGVHSM 110
    ΔTMEM242    1  -------------------------------------------------------  42

    WT        111  NDFRSKMQSIFPTIPKNSESAVEWEETLKSK 141
    ΔTMEM242    1  -------------------------------  42

C   TMEM242     AGATTGCACAGGAGGAGGCGGTAGCGGAACCTTTCTCCTCCGCCTGGTTCTCGCTTCCAC
    ΔTMEM242    AGATTGCACAGGAGGAGGCGGTAGCGGAACCTTTCTCCTCCGCCTGGTTCTCG-------

    TMEM242     CCGGGCCCAGTTGGGGACGGACGCACTAGAGGCGACCTAAACATGGAGACAGCGGGCGCT
    ΔTMEM242    ----------------------CACTAGAGGCGACCTAAACATGGAGACAGCGGGCGCT

    TMEM242     GCAACTGGGCAGCCGGCCTCTGGGCTGGAGGCTCCGGGGTCCACGAATGACCGGCTTTTC
    ΔTMEM242    GCAACTGGGCAGCCGGCCTCTGGGCTGGAGGCTCCGGGGTCCACGAATGACCGGCTTTTC

    TMEM242     CTGGTTAAAGGTAAGATGTGCTGTGGTGAGGTGCGGGCGTCGGGGTGAGTTAACCCCGGA
    ΔTMEM242    CTG-------GTAAGATGTGCTGTGGTGAGGTGCGGGCGTCGGGGTGAGTTAACCCCGGA

    WT          1  METAGAATGQPASGLEAPGSTNDRLFLVKGGIFLGTVAAAGMLAGFITTLSLAKK  55
    ΔTMEM242    1  METAGAATGQPASGLEAPGSTNDRLFLVEFSLVPLLQREC---------------  40
                                              ************
    WT         56  KSPEWFNKGSMATAALPESGSSLALRALGWGSLYAWCGVGVISFAVWKALGVHSM 110
    ΔTMEM242    1  -------------------------------------------------------  40

    WT        111  NDFRSKMQSIFPTIPKNSESAVEWEETLKSK 141
    ΔTMEM242    1  -------------------------------  40

D   TMEM242     AGATTGCACAGGAGGAGGCGGTAGCGGAACCTTTCTCCTCCGCCTGGTTCTCGCTTCCAC
    ΔTMEM242    AGATTGCACAGGAGGAGGCGGTAGCGGAACCTTTCTCCTCCGCCTGGTTCTCGCTTCCAC

    TMEM242     CCGGGCCCAGTTGGGGACGGACGCACTAGAGGCGACCTAAACATGGAGACAGCGGGCGCT
    ΔTMEM242    CCGGG-------------------------------------------------------

    TMEM242     GCAACTGGGCAGCCGGCCTCTGGGCTGGAGGCTCCGGGGTCCACGAATGACCGGCTTTTC
    ΔTMEM242    ------------------------------------------------------------

    TMEM242     CTGGTTAAAGGTAAGATGTGCTGTGGTGAGGTGCGGGCGTCGGGGTGAGTTAACCCCGGA
    ΔTMEM242    -----TAAAGGTAAGATGTGCTGTGGTGAGGTGCGGGCGTCGGGGTGAGTTAACCCCGGA

E   TMEM242     AGATTGCACAGGAGGAGGCGGTAGCGGAACCTTTCTCCTCCGCCTGGTTCTCGCTTCCAC
    ΔTMEM242    AGATTGCACAGGAGGAGGCGGTAGCGGAACCTTTCTCCTCCGCCTGGTTCTCGTCCACCC

    TMEM242     CCGGGCCCAGTTGGGGACGGACGCACTAGAGGCGACCTAAACATGGAGACAGCGGGCGCT
    ΔTMEM242    CCGGGCCCAG--------------------------------------------------

    TMEM242     GCAACTGGGCAGCCGGCCTCTGGGCTGGAGGCTCCGGGGTCCACGAATGACCGGCTTTTC
    ΔTMEM242    ------------------------------------------------------------

    TMEM242     CTGGTTAAAGGTAAGATGTGCTGTGGTGAGGTGCGGGCGTCGGGGTGAGTTAACCCCGGA
    ΔTMEM242    -----TAAAGGTAAGATGTGCTGTGGTGAGGTGCGGGCGTCGGGGTGAGTTAACCCCGGA
```

Fig. S10. The sequences of disrupted *TMEM70* and alleles of *TMEM242* in HEK293-Δδ and HEK293-Δδ.ΔTMEM70 cells. (*A*) alignment of sequences of exon I of *TMEM70* in HEK293 cells and in clonal HEK293-Δδ.ΔTMEM70 cells. The deletion includes the translational initiator codon; (*B*-*E*) three different modified alleles were identified in HEK293-Δδ.ΔTMEM242 clonal cells, and one modified gene in the HEK293-Δδ.ΔTMEM70.ΔTMEM242 clonal cells. In the upper part of panels (*B*-*E*), alignment of the sequences of the targeted gene region with the intact sequence of *TMEM242*. Carets indicate the PAM (protospacer adjacent motif) sequences for each guide RNA, and solid lines the target sequences for guide RNAs. In the wild type *TMEM242*, exon I and parts of the 5' upstream and downstream intron A (grey boxes) are shown. The arrow indicates the translational initiation codons in exon I, and deleted nucleotides are denoted by dashed lines. In the lower parts of panels (*B* and *C*), mutated protein sequences are displayed beneath the parental wild-type sequence. Asterisks, denotes amino acid changes; dashes, deleted amino acids. In panels (*D* and *E*), the deletion removed most of exon I including the translational initiator methionine codon, and part of the 5'-upstream sequences, and therefore presumably no protein was produced.

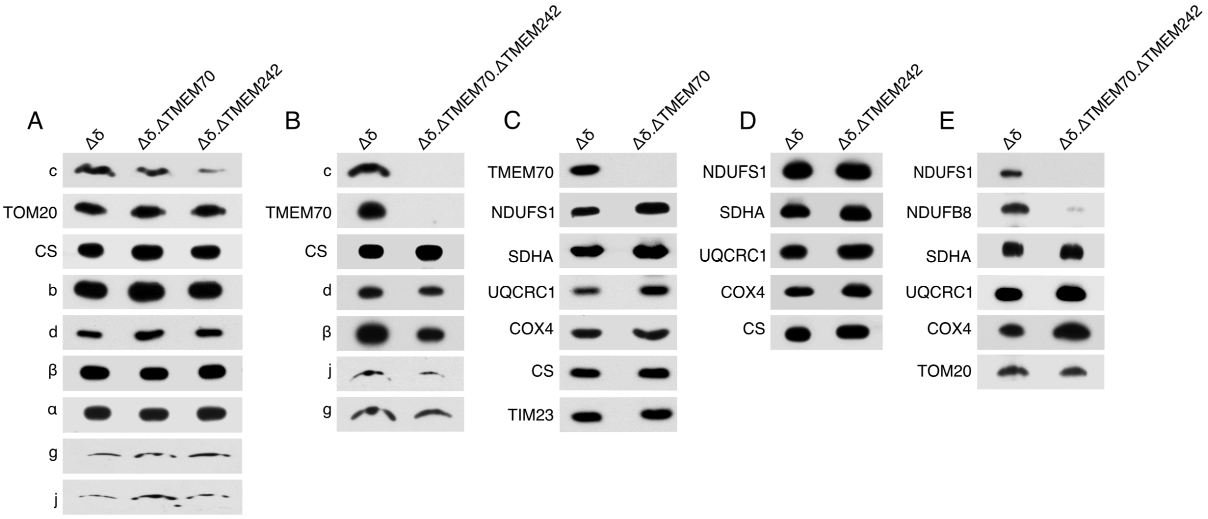

Fig. S11. Effect of the deletion of TMEM70 or TMEM242 on levels of subunits of ATP synthase and respiratory complexes in HEK293-Δδ cells. SDS-PAGE of DDM extracts of mitoplasts from HEK293-Δδ, HEK293-Δδ.ΔTMEM70, HEK293-Δδ.ΔTMEM242 and HEK293-Δδ.ΔTMEM70.ΔTMEM242 clonal cells, shown above each panel. Proteins indicated on the left were detected with antibodies. (*A* and *B*), impact on levels of subunits of ATP synthase; (*C*-*E*) representative subunits of respiratory complexes I, III and IV. Citrate synthase (CS), TOM20 and TIM23 served as the loading controls.

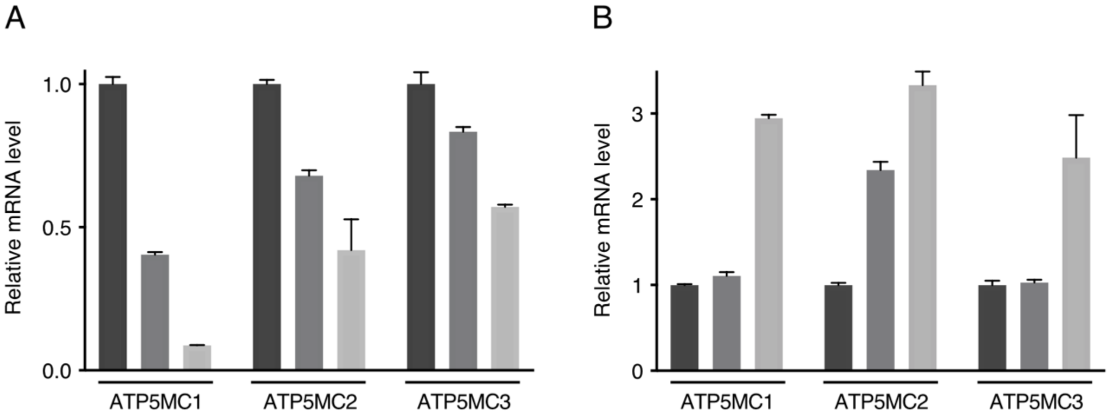

Fig. S12. Relative levels of transcripts from *ATP5MC1, ATP5MC2 and ATP5MC3* encoding subunit c of ATP synthase in HAP1 and HEK293-Δδ cells. The levels of the mRNAs for the c-subunit relative to that for β-actin are shown as the mean plus the standard error of the mean (n=3). (*A*), HAP1-WT and derivative cells; (*B*) HEK293-Δδ and derivative cells. ■ HAP1-WT or HEK293-Δδ cells; ■ HAP1-ΔTMEM242 or HEK293-Δδ.ΔTMEM242 cells; ■ HAP1-ΔTMEM70.ΔTMEM242 or HEK293-Δδ.ΔTMEM70.ΔTMEM242 cells.

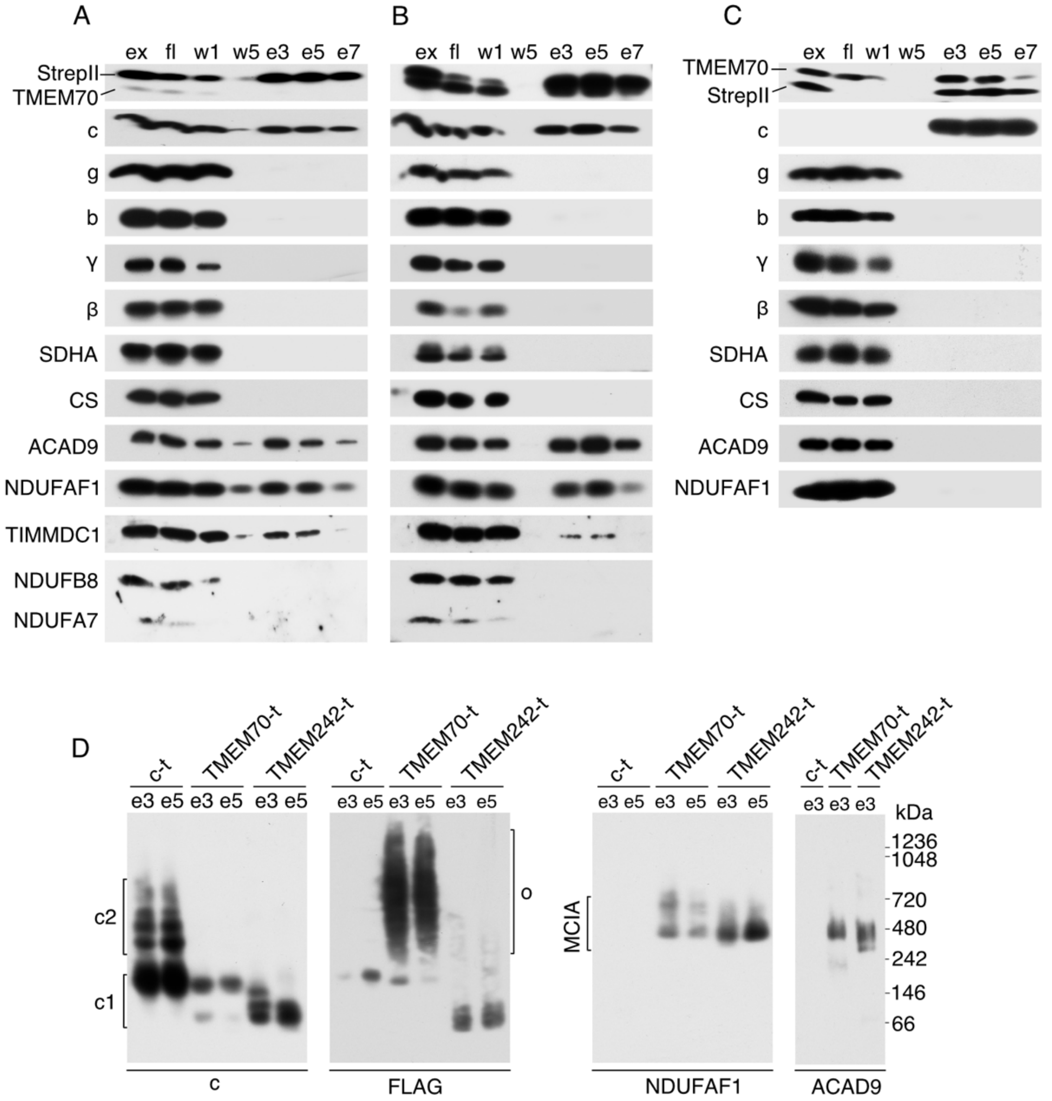

Fig. S13. Interaction of TMEM70 and TMEM242 with the c-subunit of ATP synthase and with the MCIA complex. (*A-C*), SDS-PAGE analysis of the following: (*A* and *B*) TMEM70-t and TMEM24-t, respectively, expressed in HEK293 cells, (*C*) c-t expressed in HEK293-Δδ cells. TMEM70-t, TMEM242-t and subunit c-t were detected with an anti-Strep II antibody, and other proteins with the specific antibodies indicated on the left of the panels. The top panels in *A-C* were probed twice, once with a Strep II antibody and then with an antibody for TMEM70. Extracts of mitochondria (ex) with digitonin (digitonin:protein, 10:1, w/w) were applied under gravity to a Strep II column, and the flow-through (fl) collected. The column was washed five times with 1 bed vol of buffer (w1-w5). Bound proteins were eluted with seven further portions of 0.5 bed vols of elution buffer (e1-e7) containing 10 mM desthiobiotin; (*D*) BN-PAGE analysis of eluates e3 and e5; complexes were detected with specific antibodies against the proteins indicated beneath the panels, and subunit c-t, TMEM70-t and TMEM242-t with an anti-FLAG antibody. c1, complexes containing subunit c-t and TMEM70-t and/or TMEM242-t; c2, complexes of subunit c-t lacking TMEM70-t and TMEM242-t; o, oligomers of TMEM70; MCIA, mitochondrial complex I assembly complex. The positions of molecular mass markers are indicated on the right.

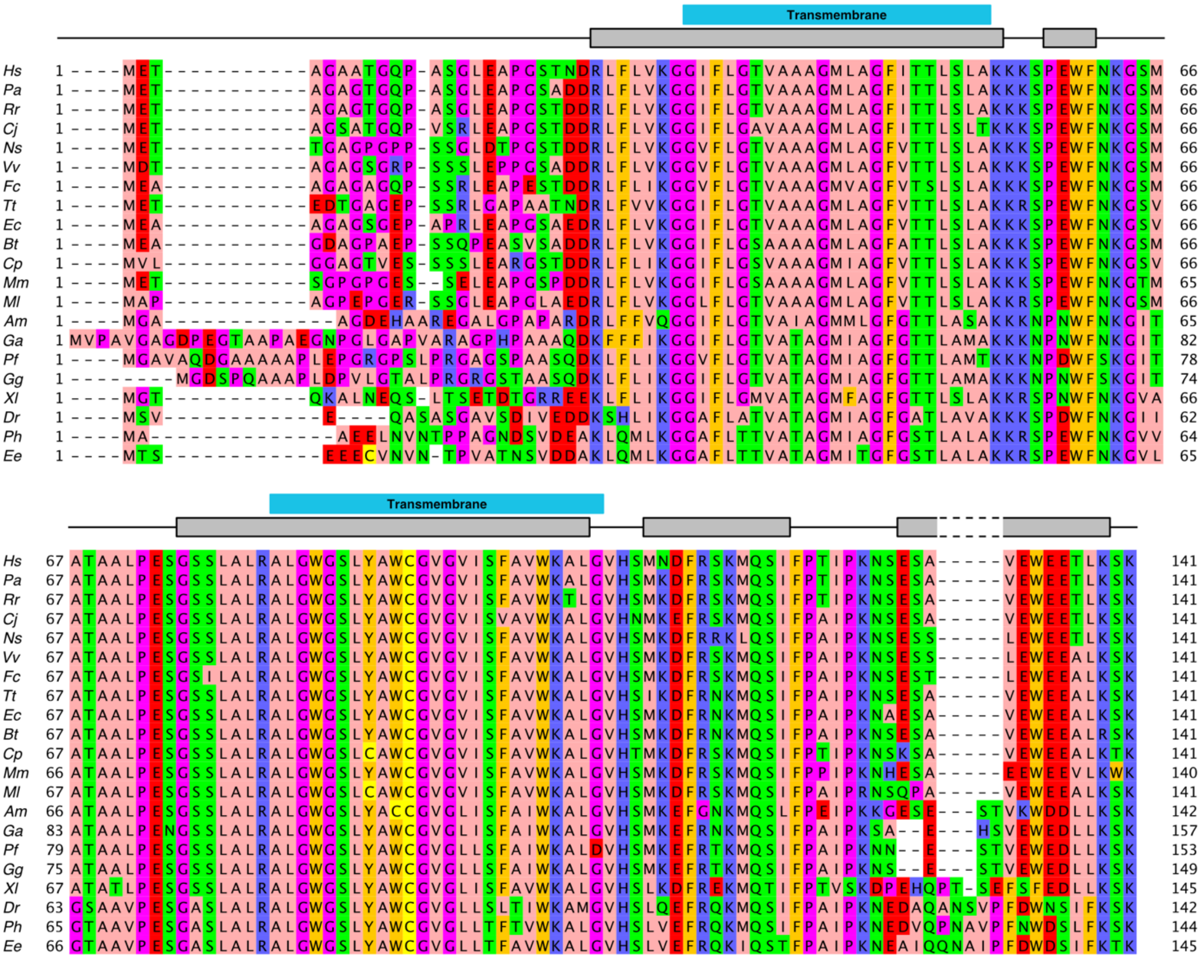

Fig. S14. Alignment of sequences of TMEM242 from various vertebrates. The alignment was performed with Clustal Omega (1.2.4) (17) and the sequences are displayed with Jalview (18) with the following color scheme: pink, aliphatic or hydrophobic; orange, aromatic; blue, positive; red, negative; green, hydrophilic; magenta, special conformation; yellow, Cys). The predicted secondary structure [PSIPRED 4 (19)] and maximal transmembrane regions [HMMTOP (20), TMHMM2.0 (21) and MEMSAT-SVM (22)] for human TMEM242 are depicted above the aligned sequences where the line denotes random coil, grey bars helices, and cyan bars transmembrane regions. The sequence data were taken from Swiss-Prot (sp), TrEMBL (tr) and NCBI (ncbi) databases. Hs, *Homo sapiens* (Human, sp|Q9NWH2); Pa, *Pongo abelii* (Sumatran orangutan, sp|Q5R987); Rr, *Rhinopithecus roxellana* (Golden snub-nosed monkey, tr|A0A2K6RSK0); Cj, *Callithrix jacchus* (Common marmoset, tr|U3DP71); Ns, *Neomonachus schauinslandi* (Hawaiian monk seal, tr|A0A2Y9HH91); Vv, *Vulpes vulpes* (Red fox, tr|A0A3Q7TBG2); Fc, *Felis catus* (Cat, tr|A0A2I2UIW5); Tt, *Tursiops truncates* (Common bottlenose dolphin, tr|A0A2U4CNL3); Ec, *Equus caballus* (Horse, tr|F6W8V8); Bt, *Bos taurus* (Cow, tr|F6QST6); Cp, *Cavia porcellus* (Guinea pig, tr|A0A286XPI8); Mm, *Mus musculus* (Mouse, sp|Q8VCR3); Ml, *Myotis lucifugus* (Little brown bat, ncbi|XP_006093938); Am, *Alligator mississippiensis* (American alligator, tr|A0A151M2F0); Ga, *Gopherus agassizii* (Desert tortoise, tr|A0A452J0W4); Pf, *Patagioenas fasciata monilis* (Band-tailed pigeon, tr|A0A1V4JLG6); Gg, *Gallus gallus* (Chicken, ncbi|XP_419691); Xl, *Xenopus laevis* (African clawed frog, sp|Q63ZZ0); Dr, *Danio rerio* (Zebrafish, sp|Q4V8S3); Ph, *Pangasianodon hypophthalmus* (Iridescent shark, tr|A0A5N5LB59); Ee, *Electrophorus electricus* (Electric eel, tr|A0A4W4G3X0).

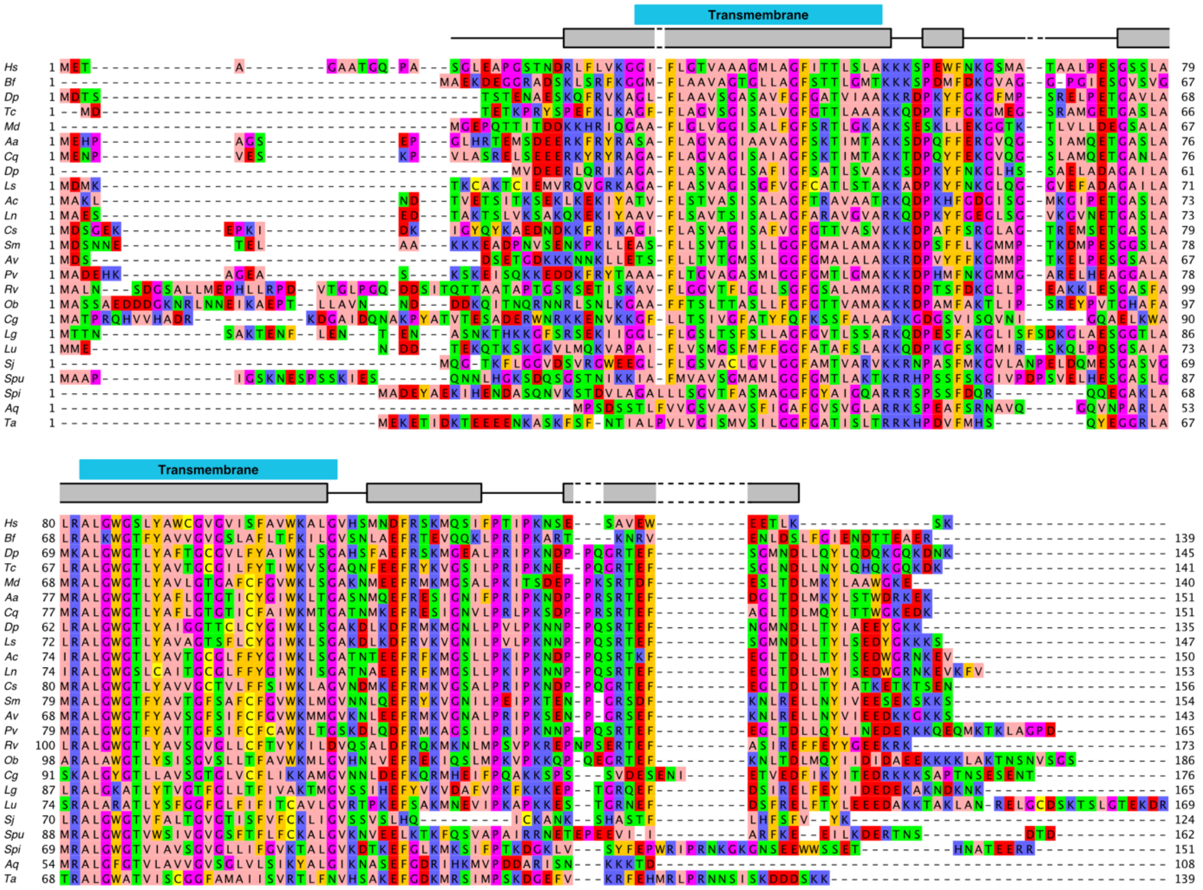

Fig. S15. Alignment of sequences of human TMEM242 with invertebrate orthologs. The predicted secondary structure [PSIPRED 4 (19)] and maximal transmembrane regions [HMMTOP (20), TMHMM2.0 (21) and MEMSAT-SVM (22)] of human TMEM242 are depicted above the aligned sequences; line denotes random coil, grey bars are helix, and cyan bars are transmembrane regions. Sequence data from Swiss-Prot (sp) and TrEMBL (tr) databases. Sequences were aligned with Clustal Omega (1.2.4) (17) and displayed with Jalview (18) using the Zappo color scheme for amino acids – see Fig. S14 legend). Hs, *Homo sapiens* (human, sp|Q9NWH2); Bf, *Branchiostoma floridae* (Florida lancelet, tr|C3ZLF6); Dp, *Dendroctonus ponderosae* (Mountain pine beetle, tr|U4UGP2); Tc, *Tribolium castaneum* (Red flour beetle, tr|D6WMN6); Md, *Musca domestica* (House fly, tr|A0A1I8MMV8); Aa, *Aedes aegypti* (Yellow fever mosquito, tr|Q17G03); Cq, *Culex quinquefasciatus* (Southern house mosquito, tr|B0X7P0); Dp, *Danaus plexippus* (Monarch Butterfly, tr|A0A212EV61); Ls, *Leptidea sinapis* (Wood white butterfly, tr|A0A5E4R0V7); Ac, *Atta cephalotes* (Leafcutter ant, tr|F6QST6); Ln, *Lasius niger* (Black garden ant, tr|A0A0J7KLI5); Cs, *Cryptotermes secundus* (Drywood termite, tr|A0A2J7Q7L5); Sm, *Stegodyphus mimosarum* (African social velvet spider, tr|A0A087UBR2); Av, *Araneus ventricosus* (Orbweaver spider, tr|A0A4Y2FS97); Pv, *Penaeus vannamei* (Whiteleg shrimp, tr|A0A423TCG2); Rv, *Ramazzottius varieornatus* (Tardigrade-water bear, tr|A0A1D1VTZ7); Ob, *Octopus bimaculoides* (California two-spotted octopus, tr|A0A0L8HN15); Cg, *Crassostrea gigas* (Pacific oyster, tr|K1RK00); Lg, *Lottia gigantea* (Giant owl limpet, tr|V3ZWT9); Lu, *Lingula unguis* (Common oriental lamp shell, tr|A0A1S3ISN3); Sj, *Stichopus japonicus* (Japanese sea cucumber, tr|A0A2G8KYE0); Spu, *Strongylocentrotus purpuratus* (Purple sea urchin, tr|W4XCD9); Spi, *Stylophora pistillata* (Smooth cauliflower coral, tr|A0A2B4SZ46); Aq, *Amphimedon queenslandica* (Sponge, tr|A0A1X7UZ91); Ta, *Trichoplax adhaerens* (Trichoplax, tr|B3RXS8).

A

```
TMEM242    1 METAGAATGQPASGLEAPGSTNDRLFLVKGGIFLGTVAAAGML-AGFITTLS-LAKKKSPEWFNKGSMAT  68
TMEM70    82 --------------LNTPSDKSEDGRLI----YTGNMARAVFGVKCFSYSTSLIGLTFLPYIFTQNNAIS 133
                           *::*....:   *:    : *.:* * :    *  : * :. .  *  *.:..  :

TMEM242   69 AALPESGSSLALRALGWGSLYAWCGVGV--ISF---------------AVWKALGVH------------- 108
TMEM70   134 ESVP-----LPIQIIFYGIMGSFTVITPVLLHFITKGYVIRLYHEATTDTYKAITYNAMLAETSTVFHQN 198
              ::*     * :: : :* : ::  :    : *                .:**:  :

TMEM242  109 ---------SMNDFRSKMQSIF--PTIPKNSESAV-----------E-WEETLKSK------ 141
TMEM70   199 DVKIPDAKHVFTTFYAKTKSLLVNPVLFPNREDYIHLMGYDKEEFILYMEETSEEKRHKDDK 260
                       :. * :* :*::  *.:  * *. :              *** :.*
```

B

```
TMEM242    1 METAGAATGQPASGLEAPGSTNDRLFLVKGGIFLGTVAAAGMLAGFITTLSLAKKKSPEWFNKGSMATAA  70
PSIPRED      -----------------------HHHHHHHHHHHHHHHHHHHHHHHHHHHHHHH---HHHH---------
JPRED        -----------------------HHHHHHHHHHHHHHHHHHHHHHHHHHHHHHH----------------

TMEM242   71 LPESGSSLALRALGWGSLYAWCGVGVISFAVWKALGVHSMNDFRSKMQSIFPTIPKNSESAVEWEETLKSK 141
PSIPRED      ----HHHHHHHHHHHHHHHHHHHHHHHHHHHHHHH----HHHHHHHHHHH--------HHHHHHHHHHH--
JPRED        ---HHHHHHHHHHH--HHHHHHHHHHHHHHHHHHH----HHHHHHHHHHH-------------HHH-----
```

C

```
TMEM70    82 LNTPSDKSEDGRLIYTGNMARAVFGVKCFSYSTSLIGLTFLPYIFTQNNAISESVPLPIQIIFYGIMGSF 151
PSIPRED      -----------EEEEEE--HHHHHHHHHHHHHHHHHHHHHHHHHHHH---------HHHHHHHHHHHHHH
JPRED        -----------EEEEEE----HHHHHHHHHHHHHHHHHHHHHHHHHH---------HHHHHHHHHHHHHH

TMEM70   152 TVITPVLLHFITKGYVIRLYHEATTDTYKAITYNAMLAETSTVFHQNDVKIPDAKHVFTTFYAKTKSLLV 221
PSIPRED      HHHHHHHHHHHH---EEEEEE-----EEEEEEEE----EEEEEEEHHH---------EEEEEE--EEEEE
JPRED        HHHHHHHHHHHH---EEEEEEE----EEEEEEE-----EEEEEEEE--EEE------EEEEEE---EEEE

TMEM70   222 NPVLFPNREDYIHLMGYDKEEFILYMEETSEEKRHKDDK 260
PSIPRED      -HHH---HHHHHHHH----HHHHHHHHH--HHH------
JPRED        -------HHHHHHHH------------------------
```

Fig. S16. Comparison of the protein sequences of human TMEM242 and TMEM70. (*A*) comparison of the protein sequences of human TMEM242 and mature human TMEM70 (Q9BUB7) with Clustal Omega v 1.2.4. The symbols *, :, and . denote identical, strongly conserved, and weakly conserved residues, respectively. The N-terminal methionine residue of TMEM242 is N-acetylated (Uniprot Q9NWH2). (*B* and *C*), secondary structure predictions for TMEM242 and mature TMEM70, respectively, produced with PSIPRED 4.0 (19) and JPred 4 (23), where dashes, H, and E denote random coil, α-helix, and β-strand, respectively, and grey boxes transmembrane α-helical regions predicted by a combination of analyses from HMMTOP (20), TMHMM (21) and MEMSAT-SVM in the PSIPRED server (22).

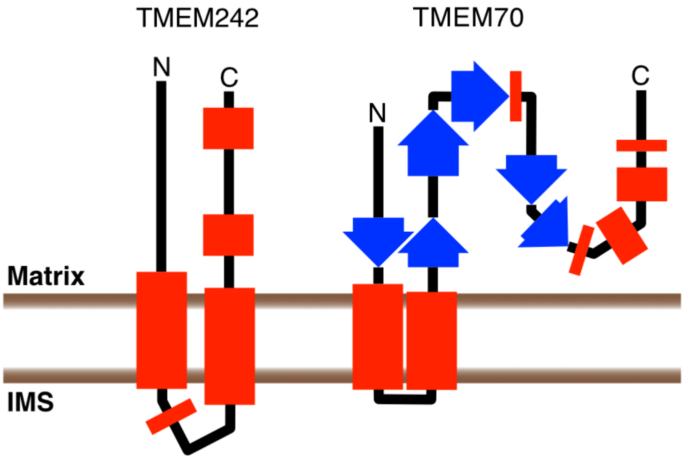

Fig. S17. Topologies and secondary structures of TMEM242 and TMEM70. The brown horizontal lines denote the boundaries of the IMM. Red rods and blue arrows represent α-helices and β-strands, respectively. Random coils at the N- and C-termini and between α-helices and β-strands are depicted as black lines. The predicted secondary structures are taken from Fig. S16. The topology of TMEM70 has been described previously (24).

Table S1. Target sites for gRNAs for the disruption of *TMEM70* and *TMEM242*

gRNA	Sequence
TMEM70-1 [1]	CGGCTGGAGTACGGGGCCTT
TMEM70-2 [2]	GAAGCCGTGTCTCGCAGTCG
TMEM70-3 [2,3]	GAGGCAGTTCGACCGCCCAC
TMEM242-1 [3]	TAGTGCGTCCGTCCCCAACT
TMEM242-2	GACCGGCTTTTCCTGGTTAA

Employed in [1] HAP1 cells and [2] HEK293 cells and derivatives thereof; [3] antisense oligonucleotide used.

Table S2. Primers for the amplification by PCR of regions in *TMEM70* and *TMEM242* targeted by gRNAs

Primer	Sequence
TMEM70-Forward	AATTCTAGGAAGGAGGAGCCG
TMEM70-Reverse	AGTAAGCACTTTCCAGCCGTT
TMEM242-Forward	AGATCCTCCCTAAGAGACGGC
TMEM242-Reverse	TGACGGGGAAATATGGCACG

Table S3. Sources of Antibodies

Protein	Source	Antibody
TMEM70	Santa Cruz	sc-393619
FLAG	Sigma	F1316
TOM20	Santa Cruz	sc-11415
NDUFS1	Proteintech	12444-1-AP
NDUFB8	Sigma	HPA003886
NDUFA7	In-house	chicken; against N-α-acetyl-residues 1-18[a]
ACAD9	Sigma	HPA037716
NDUFAF1	Abcam	ab79826
TIMMDC1	In-house	chicken; against C-terminal 12 residues[b]
ATAD3-C	In-house	rabbit; against residues 264-617[c]
ATP5F1D	ABclonal	A9929
ATP5F1E	Abcam	ab54879
p53	Cell Signaling Technology	2527
H2A	Abcam	ab18255
vinculin	Sigma	V9131

Peptide antigens; [a] Ac-ASATRLIQRLRNWASGHD; [b] RNPSVIDKQDKD; [c] amino acid sequence of human ATAD3B isoform.

Legends for Datasets S1 to S25

Dataset S1 (separate file). Proteins identified in SILAC experiments comparing affinity purified subunit c-t and subunit g-t expressed in HEK293-Δδ cells. This information is the output from Perseus after processing of MaxQuant SILAC peptide pair data. Samples were affinity purified from mitoplasts prepared from a 1:1 mixture of Flp-In™ T-REx™ HEK293-Δδ cells overexpressing either subunit c-t or subunit g-t, and fractionated by SDS-PAGE. Gel sections were digested with trypsin or chymotrypsin. Experiment 1 refers to heavy isotope labelled subunit c-t cells mixed with light labelled subunit g-t cells, and experiment 2 is vice versa. Perseus processing removed proteins identified in both a decoy database (created in MaxQuant by reversing protein entries) and a contaminant database, experiment 2 ratios were inverted, and the protein ratios rendered as base two logarithmic values. Only protein groups (listed under "Protein names") with ratios determined in both experiments are included. The protein ratio is derived from a minimum of two peptide ratios from each experiment. Ratios for subunits c-t and g-t, and endogenous subunit c and subunit g were calculated manually with data from different migration positions on the gel (see Datasets S2 and S3).

Dataset S2 (separate file). Peptide data for ATP synthase subunit c obtained in SILAC experiments comparing affinity purified subunit c-t and subunit g-t expressed in HEK293-Δδ cells. This information was obtained from the MaxQuant evidence file after the processing of SILAC peptide pair data. Samples were affinity purified from digitonin solubilized mitoplasts prepared from a 1:1 mixture of Flp-In™ T-REx™ HEK293-Δδ cells overexpressing either subunit c-t or subunit g-t. Peptide data are derived from chymotrypsin digests. Experiment 1 refers to heavy isotope labelled subunit c-t cells mixed with light labelled subunit g-t cells, and experiment 2 is vice versa. Only peptide data that provided ratio information are included. Gel section 10 corresponds to peptides from subunit c-t, and gel section 12 to peptides from endogenous subunit c. When fewer than three MULTI-MSMS ratios were obtained, ISO-MSMS ratios were included also so as to determine the protein ratio, represented by the median peptide ratio.

Dataset S3 (separate file). Peptide data for ATP synthase subunit g obtained in SILAC experiments comparing affinity purified subunit c-t and subunit g-t expressed in HEK293-Δδ cells. This information was obtained from the MaxQuant evidence file after the processing of SILAC peptide pair data. Samples were affinity purified from digitonin solubilized mitoplasts prepared from a 1:1 mixture of Flp-In™ T-REx™ HEK293-Δδ cells overexpressing either subunit c-t or subunit g-t. Peptide data were derived from both trypsin and chymotrypsin digests. Experiment 1 refers to heavy isotope labelled subunit c-t cells mixed with light labelled subunit g-t cells, and experiment 2 is vice versa. Only peptide data that provided ratio information are included. Gel sections 9 and 10 correspond to peptides from subunit g-t, and gel section 11 to the peptides from the endogenous subunit g. When fewer than three MULTI-MSMS ratios were obtained, ISO-MSMS ratios were included also so as to determine the protein ratio, represented by the median peptide ratio.

Dataset S4 (separate file). Proteins identified in SILAC experiments comparing immunopurified ATP synthase from HAP1-WT and and HAP1-ΔTMEM70 cells. This information is the output from Perseus after processing of MaxQuant SILAC peptide pair data. ATP synthase was immunopurified from digitonin solubilized mitoplasts prepared from a 1:1 mixture of HAP1-WT and HAP1-ΔTMEM70 cells, and digested in-solution with either trypsin or chymotrypsin. Experiment 1 refers to heavy isotope labelled HAP1-ΔTMEM70 cells mixed with light labelled WT cells, and experiment 2 is vice versa. Perseus processing removed proteins identified in both a decoy database (created in MaxQuant by reversing protein entries) and a contaminant database. Experiment 2 ratios were inverted, and the protein ratios rendered as base two logarithmic values. Only protein groups (listed under "Protein names") with ratios determined in both experiments are included. The protein ratio is derived from a minimum of two peptide ratios from each experiment, except for the ATP synthase subunit c protein ratio for both experiments, which was obtained from a single peptide value. ATP5IF1 ratios were calculated manually with data for unique peptides from the N-terminus of one mature form (IF_1-M1) of the protein (see Dataset S5).

Dataset S5 (separate file). Peptide data for IF_1 obtained in SILAC experiments comparing immunopurified ATP synthase from HAP1-WT and HAP1-ΔTMEM70 cells. This information was obtained from the MaxQuant evidence file after the processing of SILAC peptide pair data. ATP synthase was immunopurified from digitonin solubilized mitoplasts prepared from a 1:1 mixture of HAP1 WT and HAP1-ΔTMEM70 cells. Experiment 1 refers to heavy isotope labelled HAP1-ΔTMEM70 cells

mixed with light labelled WT cells, and experiment 2 is vice versa. Only peptide data that provided ratio information is included. When fewer than three MULTI-MSMS ratios were obtained ISO-MSMS ratios were included also so as to determine the protein ratio, represented by the median peptide ratio.

Dataset S6 (separate file). Proteins identified in SILAC experiments comparing mitoplasts from HAP1-WT and HAP1-ΔTMEM70 cells. This information is the output from Perseus after processing MaxQuant SILAC peptide pair data. Digitonin solubilized mitoplasts were prepared from a 1:1 mixture of HAP1-WT and HAP1-ΔTMEM70 cells, fractionated by SDS-PAGE and gel sections were digested with trypsin. Experiment 1 refers to heavy isotope labelled HAP1-ΔTMEM70 cells mixed with light labelled WT cells, and experiment 2 is vice versa. Perseus processing removed proteins identified in both a decoy database (created in MaxQuant by reversing protein entries) and a contaminant database, experiment 2 ratios were inverted, and the protein ratios rendered base two logarithmic. Only protein groups (listed under "Protein names") with ratios determined in both experiments are included. The protein ratio was derived from a minimum of two peptide ratios from each experiment, except for the TMEM242 protein ratio in experiment 2, which was obtained from a single peptide value. ATP5IF1 ratios were calculated manually with data from unique peptides at the N-terminus of one mature form, IF_1-M1 (see Dataset S7). Subunit 11 of the cytochrome bc_1 complex (UQCRFS1 mitochondrial targeting sequence) and Rieske subunit ratios were determined individually by manual calculation of the peptide data for each component (see Dataset S8), and FMC1 specific peptide data were employed in the manual recalculation of the protein ratio (see Dataset S9).

Dataset S7 (separate file). Peptide data for IF_1 obtained in SILAC experiments comparing mitoplasts from HAP1-WT and HAP1-ΔTMEM70 cells. This information was obtained from the MaxQuant evidence file after the processing of SILAC peptide pair data. The peptide data are from digitonin protein extracts of mitoplasts prepared with a 1:1 mixture of HAP1-WT and HAP1-ΔTMEM70 cells. Experiment 1 refers to heavy isotope labelled HAP1-ΔTMEM70 cells mixed with light labelled WT cells, and experiment 2 is vice versa. Only peptide data that provided ratio information are included.

Dataset S8 (separate file). Peptide data for the Rieske subunit and subunit 11 of cytochrome bc_1 obtained in SILAC experiments comparing mitoplasts from HAP1-WT and HAP1-ΔTMEM70 cells. This information is obtained from the MaxQuant evidence file after the processing of SILAC peptide pair data. The peptide data are from digitonin protein extracts of mitoplasts prepared with a 1:1 mixture of HAP1-WT and HAP1-ΔTMEM70 cells. Experiment 1 refers to heavy isotope labelled HAP1-ΔTMEM70 cells mixed with light labelled HAP1-WT cells, and experiment 2 is vice versa. Only peptide data that provided ratio information are included. When fewer than three MULTI-MSMS ratios were obtained, ISO-MSMS ratios are included also so as to determine the protein ratio, represented by the median peptide ratio.

Dataset S9 (separate file). Peptide data for protein FMC1 homolog obtained in SILAC experiments comparing mitoplasts from HAP1-WT and HAP1-ΔTMEM70 cells. This information is obtained from the MaxQuant evidence file after the processing of SILAC peptide pair data. The peptide data are from digitonin protein extracts of mitoplasts prepared with a 1:1 mixture of HAP1-WTand HAP1-ΔTMEM70 cells. Experiment 1 refers to heavy isotope labelled HAP1-ΔTMEM70 cells mixed with light labelled HAP1-WT cells, and experiment 2 is vice versa. Only peptide data that provided ratio information are included.

Dataset S10 (separate file). Proteins identified in SILAC experiments comparing immunopurified ATP synthase from HAP1-WT and HAP1-ΔTMEM242 cells. This information is the output from Perseus after processing of MaxQuant SILAC peptide pair data. ATP synthase was immunopurified from digitonin solubilized mitoplasts prepared from a 1:1 mixture of HAP1-WT and HAP1-ΔTMEM242 cells, and digested in-solution with either trypsin or chymotrypsin. Experiment 1 refers to heavy isotope labelled HAP1-ΔTMEM242 cells mixed with light labelled HAP1-WT cells, and experiment 2 is vice versa. Perseus processing removed proteins identified in both a decoy database (created in MaxQuant by reversing protein entries) and a contaminant database. Experiment 2 ratios were inverted, and the protein ratios rendered as base two logarithmic values. Only protein groups (listed under "Protein names") with ratios determined in both experiments are included. The protein ratio was derived from a minimum of two peptide ratios from each experiment, except for MT-ATP6 (experiment 1) where the protein ratio was from a single peptide value. ATP5IF1 ratios were calculated manually with data for unique peptides from the N-terminus of one mature form (IF_1-M1) of the protein (see Dataset S11). The

TMEM70 experiment 1 ratio was re-calculated with the median peptide value, replacing the ‘plateau’ value from MaxQuant (see Datasheet S12).

Dataset S11 (separate file). Peptide data for IF_1 obtained in SILAC experiments comparing immunopurified ATP synthase from HAP1-WT and HAP1-ΔTMEM242 cells. This information was obtained from the MaxQuant evidence file after the processing of SILAC peptide pair data. ATP synthase was immunopurified from digitonin solubilized mitoplasts prepared from a 1:1 mixture of HAP1-WTand HAP1-ΔTMEM242 cells. Experiment 1 refers to heavy isotope labelled HAP1-ΔTMEM242 cells mixed with light labelled wild-type cells, and experiment 2 is vice versa. Only peptide data that provided ratio information are included.

Dataset S12 (separate file). Peptide data for TMEM70 obtained in SILAC experiments comparing immunopurified ATP synthase from HAP1-WT and HAP1-ΔTMEM242 cells. This information was obtained from the MaxQuant evidence file after the processing of SILAC peptide pair data. ATP synthase was immunopurified from digitonin solubilized mitoplasts prepared from a 1:1 mixture of HAP1-WT and HAP1-ΔTMEM242 cells. Experiment 1 refers to heavy isotope labelled HAP1-ΔTMEM242 cells mixed with light labelled HAP1-WT cells, and experiment 2 is vice versa. Only peptide data that provided ratio information are included. When fewer than three MULTI-MSMS ratios were obtained, ISO-MSMS ratios are included also so as to determine the protein ratio, represented by the median peptide ratio.

Dataset S13 (separate file). Proteins identified in SILAC experiments comparing mitoplasts from HAP1-WT and HAP1-ΔTMEM242 cells. This information is the output from Perseus after processing of MaxQuant SILAC peptide pair data. Digitonin solubilized mitoplasts were prepared from a 1:1 mixture of HAP1-WT and HAP1-ΔTMEM242 cells, fractionated by SDS-PAGE and gel sections were digested with trypsin. Experiment 1 refers to heavy isotope labelled HAP1-ΔTMEM242 cells mixed with light labelled HAP1-WT cells, and experiment 2 is vice versa. Perseus processing removed proteins identified in both a decoy database (created in MaxQuant by reversing protein entries) and a contaminant database, experiment 2 ratios were inverted, and the protein ratios rendered as base two logarithmic values. Only protein groups (listed under “Protein names”) with ratios determined in both experiments are included. The protein ratio was derived from a minimum of two peptide ratios from each experiment. ATP5IF1 ratios were calculated manually with data for unique peptides from the N-termini of three mature forms (IF_1-M1, -M2 and M3) of the protein (see Dataset S14). The ratios for subunit 11 (UQCRFS1 mitochondrial targeting sequence) and the Rieske subunit of the cytochrome bc_1 complex were determined individually by manually calculating the peptide data for each component (see Dataset S15).

Dataset S14 (separate file). Peptide data for IF_1 obtained in SILAC experiments comparing mitoplasts from HAP1-WT and HAP1-ΔTMEM242 cells. This information was obtained from the MaxQuant evidence file after the processing of SILAC peptide pair data. The peptide data are from digitonin protein extracts of mitoplasts prepared with a 1:1 mixture of HAP1-WT and HAP1-ΔTMEM242 cells. Experiment 1 refers to heavy isotope labelled HAP1-ΔTMEM242 cells mixed with light labelled wild-type cells, and experiment 2 is vice versa. Only peptide data that provided ratio information are included. When fewer than three MULTI-MSMS ratios were obtained, ISO-MSMS ratios are also included to determine the protein ratio, represented by the median peptide ratio.

Dataset S15 (separate file). Peptide data for Rieske subunit and subunit 11 of cytochrome bc_1 obtained in SILAC experiments comparing mitoplasts from HAP1-WT and HAP1-ΔTMEM242 cells. This information was obtained from the MaxQuant evidence file after the processing of SILAC peptide pair data. The peptide data are from digitonin protein extracts of mitoplasts prepared with a 1:1 mixture of HAP1-WT and HAP1-ΔTMEM242 cells. Experiment 1 refers to heavy isotope labelled HAP1-ΔTMEM242 cells mixed with light labelled HAP1-WT cells, and experiment 2 is vice versa. Only peptide data that provided ratio information are included. When fewer than three MULTI-MSMS ratios were obtained, ISO-MSMS ratios are also included to determine the protein ratio, represented by the median peptide ratio.

Dataset S16 (separate file). Proteins identified in SILAC experiments comparing immunopurified ATP synthase from HAP1-WT and HAP1-ΔTMEM70.ΔTMEM242 cells. This information is the output from Perseus after processing of MaxQuant SILAC peptide pair data. ATP synthase was immunopurified from digitonin solubilized mitoplasts prepared from a 1:1 mixture of HAP1-WT and

HAP1-ΔTMEM70.ΔTMEM242 cells, and digested in-solution with either trypsin or chymotrypsin. Experiment 1 refers to heavy isotope labelled HAP1-ΔTMEM70.ΔTMEM242 cells mixed with light labelled HAP1-WT cells, and experiment 2 is vice versa. Perseus processing removed proteins identified in both a decoy database (created in MaxQuant by reversing protein entries) and a contaminant database, experiment 2 ratios were inverted, and the protein ratios rendered base two logarithmic. Only protein groups (listed under "Protein names") with ratios determined in both experiments are included. The protein ratio is derived from a minimum of two peptide ratios from each experiment. ATP5IF1 ratios were calculated manually with data for unique peptides from the N-termini of one mature form (IF1-M1) of the protein (see Dataset S17).

Dataset S17 (separate file). Peptide data for IF_1 obtained in SILAC experiments comparing immunopurified ATP synthase from HAP1-WT and HAP1-ΔTMEM70.ΔTMEM242 cells. This information was obtained from the MaxQuant evidence file after the processing of SILAC peptide pair data. ATP synthase was immunopurified from digitonin solubilized mitoplasts prepared from a 1:1 mixture of HAP1-WT and HAP1-ΔTMEM70.ΔTMEM242 cells. Experiment 1 refers to heavy isotope labelled HAP1-ΔTMEM70.ΔTMEM242 cells mixed with light labelled HAP1-WT cells, and experiment 2 is vice versa. Only peptide data that provided ratio information are included. When fewer than three MULTI-MSMS ratios were obtained, ISO-MSMS ratios are included also so as to determine the protein ratio, represented by the median peptide ratio.

Dataset S18 (separate file). Proteins identified in SILAC experiments comparing mitoplasts HAP1-WT and HAP1-ΔTMEM70.ΔTMEM242 cells. This information is the output from Perseus after processing of MaxQuant SILAC peptide pair data. Digitonin solubilized mitoplast samples were prepared from a 1:1 mixture of HAP1-WT and HAP1-ΔTMEM70.ΔTMEM242 cells, fractionated by SDS-PAGE and gel sections digested with trypsin. Experiment 1 refers to heavy isotope labelled HAP1-ΔTMEM70.ΔTMEM242 cells mixed with light labelled HAP1-WT cells, and experiment 2 is vice versa. Perseus processing removed proteins identified in both a decoy database (created in MaxQuant by reversing protein entries) and a contaminant database, experiment 2 ratios were inverted, and the protein ratios rendered as base two logarithmic values. Only protein groups (listed under "Protein names") with ratios determined in both experiments are included. The protein ratio was derived from a minimum of two peptide ratios from each experiment. ATP5IF1 ratios were calculated manually with data for unique peptides from the N-termini of two mature forms (IF_1-M1 and M3) of the protein (see Dataset S19). Ratios for subunit 11 (UQCRFS1 mitochondrial targeting sequence) and the Rieske subunit of cytochrome bc_1 were determined individually by manually calculating the peptide data for each component (see Dataset S20).

Dataset S19 (separate file). Peptide data for IF_1 obtained in SILAC experiments comparing mitoplasts from HAP1-WT and HAP1-ΔTMEM70.ΔTMEM242 cells. This information was obtained from the MaxQuant evidence file after the processing of SILAC peptide pair data. The peptide data are from digitonin protein extracts of mitoplasts from a 1:1 mixture of HAP1-WT and HAP1-ΔTMEM70.ΔTMEM242 cells. Experiment 1 refers to heavy isotope labelled HAP1-ΔTMEM70.ΔTMEM242 cells mixed with light labelled HAP1-WT, and experiment 2 is vice versa. Only peptide data that provided ratio information are included. When fewer than three MULTI-MSMS ratios were obtained ISO-MSMS, ratios are included also to determine the protein ratio, represented by the median peptide ratio.

Dataset S20 (separate file). Peptide data for the Rieske subunit and subunit 11 of cytochrome bc_1 obtained in SILAC experiments comparing mitoplasts from HAP1-WT and HAP1-ΔTMEM70.ΔTMEM242 cells. This information was obtained from the MaxQuant evidence file after the processing of SILAC peptide pair data. The peptide data are from digitonin protein extracts of mitoplasts from a 1:1 mixture of HAP1-WT and HAP1-ΔTMEM70.ΔTMEM242 cells. Experiment 1 refers to heavy isotope labelled HAP1-ΔTMEM70.ΔTMEM242 cells mixed with light labelled HAP1-WT cells, and experiment 2 is vice versa. Only peptide data that provided ratio information are included. When fewer than three MULTI-MSMS ratios were obtained, ISO-MSMS ratios are included also so as to determine the protein ratio, represented by the median peptide ratio.

Dataset S21 (separate file). Proteins identified in SILAC experiments comparing mitoplasts from HEK293 parental, HEK293-Δδ and HEK293-Δδ.ΔTMEM70 cells. Relative protein abundance was determined by quantitative mass spectrometry and a 3-way SILAC labelling procedure. Heavy labelled (H; Arg-10, Lys-8), medium labelled (M; Arg-6, Lys-4) and light (L; Arg-0, Lys-0) cells were mixed

equally in two combinations (experiment 1: parental-L, Δδ-M, Δδ.ΔTMEM70-H; experiment 2: parental-H, Δδ-M, Δδ.ΔTMEM70-L) and n-dodecyl-β-D-maltoside solubilized mitoplasts were prepared from the 1:1:1 mixtures, fractionated by SDS-PAGE and gel sections were digested with trypsin or chymotrypsin. The information is the output from Perseus after processing of MaxQuant SILAC peptide data. HEK293-Δδ relative to HEK293 parental is provided by ratios M/L in experiment 1 and H/M in experiment 2, HEK293-Δδ.ΔTMEM70 relative to HEK293-Δδ by H/M in experiment 1 and M/L in experiment 2, and HEK293-Δδ.ΔTMEM70 relative to HEK293-parental by H/L in experiment 1 and H/L in experiment 2. Perseus processing removed proteins identified in both a decoy database (created in MaxQuant by reversing protein entries) and a contaminant database. Experiment 2 ratios were inverted, and the protein ratios rendered as base two logarithmic values. The protein ratio was derived from a minimum of two peptide ratios from each experiment. Specific protein ratios for two forms of ATP5F1D were re-calculated with MaxQuant applied to gel section 9 (aberrant form) and gel section 10 (endogenous form). "NaN" is "not a number", as no ratio was determined.

Dataset S22 (separate file). Proteins identified in SILAC experiments comparing mitoplasts from HEK293 parental, HEK293-Δδ and HEK293-Δδ.ΔTMEM242 cells. Relative protein abundances were determined by quantitative mass spectrometry and a 3-way SILAC labelling procedure. Heavy labelled (H; Arg-10, Lys-8), medium labelled (M; Arg-6, Lys-4) and light (L; Arg-0, Lys-0) cells were mixed equally in two combinations (experiment 1: HEK293-parental-L, HEK293-Δδ-M, HEK293-Δδ.ΔTMEM242-H; experiment 2: HEK293-parental-H, HEK293-Δδ-M, HEK293-Δδ.ΔTMEM242-L) and n-dodecyl-β-D-maltoside solubilized mitoplasts were prepared from the 1:1:1 mixtures, fractionated by SDS-PAGE and gel sections were digested with trypsin or chymotypsin. The information is the output from Perseus after processing of MaxQuant SILAC peptide data. HEK293-Δδ relative to HEK293-parental is provided by ratios M/L from experiment1 and H/M from experiment 2, HEK293-Δδ.ΔTMEM242 relative to HEK293-Δδ by H/M from experiment 1 and M/L from experiment 2, and HEK293-Δδ.ΔTMEM242 relative to HEK293-parental by H/L in experiment 1 and H/L in experiment 2. Perseus processing removed proteins identified in both a decoy database (created in MaxQuant by reversing protein entries) and a contaminant database. Experiment 2 ratios were inverted, and the protein ratios were rendered as base two logarithmic values. The protein ratio was derived from a minimum of two peptide ratios from each experiment. "NaN" is "not a number', as no ratio was determined.

Dataset S23 (separate file). Proteins identified in SILAC experiments comparing affinity purified TMEM242-t and METTL12-t expressed in HEK293 cells. This information is the output from Perseus after processing of MaxQuant SILAC peptide pair data. Samples were affinity purified from mitoplasts from a 1:1 mixture of Flp-In™ T-REx™ HEK293 cells overexpressing either TMEM242-t or METTL12-t, fractionated by SDS-PAGE and gel sections were digested with trypsin or chymotrypsin. Experiment 1 refers to heavy isotope labelled TMEM242-t cells mixed with light labelled METTL12-t cells, and experiment 2 is vice versa. Perseus processing removed proteins identified in both a decoy database (created in MaxQuant by reversing protein entries) and a contaminant database. Experiment 2 ratios were inverted, and the protein ratios were rendered as base two logarithmic values. Only protein groups (listed under "Protein names") with ratios determined in both experiments are included. The protein ratio was derived from a minimum of two peptide ratios from each experiment. ATP5IF1 ratios were calculated manually with data for unique peptides from the N-termini of one mature form (IF_1-M1) of the protein (see Dataset S24).

Dataset S24 (separate file). Peptide data for IF_1 obtained in SILAC experiments comparing affinity purified TMEM242-t and METTL12-t expressed in HEK293 cells. This information was obtained from the MaxQuant evidence file after the processing of SILAC peptide pair data. Samples were affinity purified from digitonin solubilized mitoplasts from a 1:1 mixture of Flp-In™ T-REx™ HEK293 cells overexpressing either TMEM242-t or METTL12-t. Peptide data were derived from both trypsin and chymotrypsin digests. Experiment 1 refers to heavy isotope labelled TMEM242-t cells mixed with light labelled METTL12-t cells, and experiment 2 is vice versa. Only peptide data that provided ratio information are included. When fewer than three MULTI-MSMS ratios were obtained, ISO-MSMS ratios are included also so as to determine the protein ratio, represented by the median peptide ratio.

Dataset S25 (separate file). Proteins identified in SILAC experiments comparing affinity purified TMEM70-t and METTL12-t expressed in HEK293 cells. This information is the output from Perseus after processing of MaxQuant SILAC peptide pair data. Samples were affinity purified from mitoplasts from a 1:1 mixture of Flp-In™ T-REx™ HEK293 cells overexpressing either TMEM70-t or METTL12-t,

fractionated by SDS-PAGE, and gel sections were digested with trypsin or chymotrypsin. Experiment 1 refers to heavy isotope labelled TMEM70-t cells mixed with light labelled METTL12-t cells, and experiment 2 is vice versa. Perseus processing removed proteins identified in both a decoy database (created in MaxQuant by reversing protein entries) and a contaminant database. Experiment 2 ratios were inverted, and the protein ratios rendered as base two logarithmic values. Only protein groups (listed under "Protein names") with ratios determined in both experiments are included. The protein ratio was derived from a minimum of two peptide ratios from each experiment, except for the ratio for ATP synthase subunit c for both experiments which is derived from a single peptide value.

SI References

1. V. F. Rhein, *et al.*, Human METTL20 methylates lysine residues adjacent to the recognition loop of the electron transfer flavoprotein in mitochondria. *J Biol Chem* **289**, 24640-24651 (2014).
2. P. Klement, L. G. Nijtmans, C. Van den Bogert, J. Houstěk, Analysis of oxidative phosphorylation complexes in cultured human fibroblasts and amniocytes by blue-native-electrophoresis using mitoplasts isolated with the help of digitonin. *Anal Biochem* **231**, 218-224 (1995).
3. H. Schägger, G. von Jagow, Blue native electrophoresis for isolation of membrane protein complexes in enzymatically active form. *Anal Biochem* **199**, 223-231 (1991).
4. I. Wittig, M. Karas, H. Schägger, High resolution clear native electrophoresis for in-gel functional assays and fluorescence studies of membrane protein complexes. *Mol Cell Proteomics* **6**, 1215-1225 (2007).
5. J. He, *et al.*, Persistence of the mitochondrial permeability transition in the absence of subunit c of human ATP synthase. *Proc Natl Acad Sci U S A* **114**, 3409-3414 (2017).
6. M. Wilm, *et al.*, Femtomole sequencing of proteins from polyacrylamide gels by nano-electrospray mass spectrometry. *Nature* **379**, 466-469 (1996).
7. J. He, *et al.*, Human C4orf14 interacts with the mitochondrial nucleoid and is involved in the biogenesis of the small mitochondrial ribosomal subunit. *Nucleic Acids Res* **40**, 6097-6108 (2012).
8. S. E. Ong, *et al.*, Stable isotope labeling by amino acids in cell culture, SILAC, as a simple and accurate approach to expression proteomics. *Mol Cell Proteomics* **1**, 376-386 (2002).
9. P. Skehan, *et al.*, New colorimetric cytotoxicity assay for anticancer-drug screening. *J Natl Cancer Inst* **82**, 1107-1112 (1990).
10. F. A. Ran, *et al.*, Genome engineering using the CRISPR-Cas9 system. *Nat Protoc* **8**, 2281-2308 (2013).
11. J. He, J. Carroll, S. Ding, I. M. Fearnley, J. E. Walker, Permeability transition in human mitochondria persists in the absence of peripheral stalk subunits of ATP synthase. *Proc Natl Acad Sci U S A* **114**, 9086-9091 (2017).
12. J. He, *et al.*, Assembly of the membrane domain of ATP synthase in human mitochondria. *Proc Natl Acad Sci U S A* **115**, 2988-2993 (2018).
13. J. He, *et al.*, Assembly of the peripheral stalk of ATP synthase in human mitochondria. *Proc Natl Acad Sci U S A* **117**, 29602-29608 (2020).
14. J. Cox, M. Mann, MaxQuant enables high peptide identification rates, individualized p.p.b.-range mass accuracies and proteome-wide protein quantification. *Nat Biotechnol* **26**, 1367-1372 (2008).
15. J. Cox, *et al.*, Andromeda: a peptide search engine integrated into the MaxQuant environment. *J Proteome Res* **10**, 1794-1805 (2011).
16. S. Y. Lee, *et al.*, Architecture mapping of the inner mitochondrial membrane proteome by chemical tools in live cells. *J Am Chem Soc* **139**, 3651-3662 (2017).
17. F. Sievers, *et al.*, Fast, scalable generation of high-quality protein multiple sequence alignments using Clustal Omega. *Mol Syst Biol* **7**, 539 (2011).
18. A. M. Waterhouse, J. B. Procter, D. M. Martin, M. Clamp, G. J. Barton, Jalview Version 2—a multiple sequence alignment editor and analysis workbench. *Bioinformatics* **25**, 1189-1191 (2009).
19. D. T. Jones, Protein secondary structure prediction based on position-specific scoring matrices. *J Mol Biol* **292**, 195-202 (1999).
20. G. E. Tusnády, I. Simon, The HMMTOP transmembrane topology prediction server. *Bioinformatics* **17**, 849-850 (2001).
21. A. Krogh, B. Larsson, G. von Heijne, E. L. Sonnhammer, Predicting transmembrane protein topology with a hidden Markov model: application to complete genomes. *J Mol Biol* **305**, 567-580 (2001).

22. T. Nugent, D. T. Jones, Transmembrane protein topology prediction using support vector machines. *BMC Bioinformatics* **10**, 159 (2009).
23. A. Drozdetskiy, C. Cole, J. Procter, G. J. Barton, JPred4: a protein secondary structure prediction server. *Nucleic Acids Res* **43**, W389-94 (2015).
24. H. Kratochvílová, *et al*., Mitochondrial membrane assembly of TMEM70 protein. *Mitochondrion* **15**, 1-9 (2014).
